# Macrophages as Drivers of Resistance to Dabrafenib Plus Trametinib Therapy in Anaplastic Thyroid Cancer

**DOI:** 10.3390/ijms27177691

**Published:** 2026-08-27

**Authors:** Ricardo Rodrigues, Beatriz Garcia, Miguel Rito, Rúben Roque, Teresa Pereira, Sónia Morgado, Vanessa Tavares, Tiago Nunes da Silva, Valeriano Leite, Branca Maria Cavaco

**Affiliations:** 1 Unidade de Investigação em Patobiologia Molecular (UIPM), Instituto Português de Oncologia de Lisboa Francisco Gentil (IPOLFG), Rua Professor Lima Basto, 1099-023 Lisboa, Portugal; rmrodrigues@ipolisboa.min-saude.pt (R.R.); ba.garcia@campus.fct.unl.pt (B.G.); tlnsilva@ipolisboa.min-saude.pt (T.N.d.S.); vleite@ipolisboa.min-saude.pt (V.L.); 2 Faculdade de Ciências Médicas, Universidade NOVA de Lisboa, Campo dos Mártires da Pátria, 130, 1169-056 Lisboa, Portugal; 3 Faculdade de Ciências e Tecnologia, Universidade NOVA de Lisboa, Campus da Caparica, 2829-516 Caparica, Portugal; 4 Serviço de Anatomia Patológica, Instituto Português de Oncologia de Lisboa Francisco Gentil (IPOLFG), Rua Professor Lima Basto, 1099-023 Lisboa, Portugal; mrito@ipolisboa.min-saude.pt (M.R.); rroque@ipolisboa.min-saude.pt (R.R.); tpereira@ipolisboa.min-saude.pt (T.P.); smorgado@ipolisboa.min-saude.pt (S.M.); vtavares@ipolisboa.min-saude.pt (V.T.); 5 Instituto de Anatomia Patológica, Faculdade de Medicina da Universidade de Lisboa, Av. Prof. Egas Moniz, 1649-028 Lisboa, Portugal; 6 Escola Superior de Saúde de Lisboa, Politécnico de Lisboa, Estrada de Benfica, 529, 1549-020 Lisboa, Portugal; 7 Serviço de Endocrinologia, Instituto Português de Oncologia de Lisboa Francisco Gentil (IPOLFG), Rua Professor Lima Basto, 1099-023 Lisboa, Portugal

**Keywords:** anaplastic thyroid carcinoma, Dabrafenib plus Trametinib, *SPRY4*, macrophages

## Abstract

Anaplastic thyroid carcinoma (ATC) is an aggressive malignancy with poor response to standard therapies. While Dabrafenib plus Trametinib (DT) combination therapy has improved the survival of patients with *BRAF*-mutant ATC, resistance remains a challenge. Given the association of macrophages with poor prognosis, and that *SPRY4* has emerged as a mediator in ATC–macrophage interactions, we investigated their role in DT resistance and evaluated the therapeutic potential of macrophage targeting. Transwell co-cultures of *BRAF*-mutant ATC cell lines (T235 and T238) with THP-1-derived macrophages were established, and viability, invasion, and macrophage phenotype were assessed. Macrophages were also characterised in tumour samples from eleven ATC patients treated with DT. *In vitro*, DT significantly reduced ATC cell viability and invasion. However, macrophage co-culture significantly restored these effects, also promoting cytoskeletal remodelling and increased vimentin expression, despite DT treatment. DT significantly downregulated *SPRY4* and suppressed MAPK signalling and PD-L1. These effects were also significantly reversed by macrophages. Under DT, macrophages showed an increase in M2-like polarisation in co-culture. Patients’ tumour samples were highly infiltrated with macrophages. *In vitro* targeting of macrophages with Edicotinib, in combination with DT, significantly enhanced the anti-tumour effects of DT and decreased the M2-like polarisation, bypassing the macrophage-mediated DT resistance. Overall, the *in vitro* findings suggest that macrophages modulate ATC response to DT by enhancing viability, invasion, MAPK signalling and PD-L1, supporting a pro-tumoural phenotype. Targeting macrophages overcomes this resistance, highlighting the CSF1/CSF1R signalling pathway within the ATC–macrophage axis as a promising therapeutic target to improve DT efficacy.

## 1. Introduction

Anaplastic thyroid carcinoma (ATC), although rare, is the most aggressive and lethal subtype of thyroid cancer. ATCs are characterised by a loco-regional invasiveness, which usually causes compressive symptoms, making surgery a difficult treatment option. ATCs no longer respond to radioactive iodine therapy [1,2,3]. Multikinase inhibitors, such as Lenvatinib and multimodal therapies, are associated with poor response and survival rates: for Lenvatinib, the median progression-free survival (PFS) is 2.6 months and median overall survival (OS) is 3.2 months, whereas for multimodal therapy the PFS is 4 months and OS is 3 months [2,4,5]. ATC is characterised by a highly complex molecular background with multiple gene abnormalities, in which the *BRAF* p.V600E mutations are present in approximately 50% of all ATC cases [1,2,3]. Dabrafenib plus Trametinib (DT) therapy emerged as a novel therapeutic approach for ATC patients harbouring the *BRAF* p.V600E mutation, and was approved by the FDA after a pivotal phase II clinical trial [6]. This approach led to an improvement in OS and PFS for these patients, as reported by our group and other authors [6,7,8,9,10]. Despite the remarkable clinical efficacy of BRAF/MEK inhibition in patients with *BRAF*-mutant ATC, resistance frequently limits the durability of treatment responses. Several mechanisms have been implicated in resistance to BRAF/MEK inhibitors, including reactivation of the MAPK pathway through secondary mutations in MAPK-related genes, such as the *RAS* genes, activation of alternative signalling pathways, including the PI3K/AKT/mTOR pathway, and the influence of the tumour microenvironment (TME), highlighting the need for novel therapeutic strategies to overcome DT resistance [11,12,13,14,15].

The dynamic interactions between malignant/neoplastic cells and the surrounding TME influence cancer progression and therapeutic response. ATC tumours are highly infiltrated by tumour-associated macrophages (TAMs) and exhausted CD8^+^ T cells. TAMs are particularly predominant, representing approximately 50% of the ATC tumour mass [16,17,18]. Macrophage infiltration in thyroid carcinomas (TCs) has been associated with poor prognosis, including extrathyroidal extension, as well as cervical lymph node and distant metastases. The extent of macrophage infiltration increases with the degree of tumour dedifferentiation, suggesting that macrophages may play a significant role in the progression of TC [16,19]. The association between BRAF inhibitors (BRAFi; Vemurafenib) and the TME was studied by Lee et al., who observed that, in ATC and aggressive differentiated papillary TC, upon treatment with Vemurafenib, the infiltration of activated CD8^+^ T cells decreased and the immunosuppressive behaviour of the TME increased (e.g., infiltration of M2-like macrophages) [20]. These findings highlight the relevance of macrophages in BRAFi response and the possibility of targeting macrophages in combination with BRAFi. *SPRY4* (*Sprouty RTK signalling antagonist 4*) has been implicated in cell proliferation, survival, differentiation and metastasis, and is considered an inhibitor of the MAPK pathway [21]. A recent study by our group identified this gene as a candidate tumour suppressor gene in ATC–macrophage crosstalk [22]. However, the role of *SPRY4* in the response/resistance to treatment with kinase inhibitors is unknown. Given that, macrophages seem to influence BRAFi efficacy [20] and *SPRY4* expression [22], we hypothesised that these cells may play a role in the response of ATC to DT therapy and be amenable to therapeutic modulation by alternative therapeutic strategies, while SPRY4 could also represent a mediator in this context.

Here, we investigated the role of macrophages in regulating the response of *BRAF*-mutant ATC cell lines to DT therapy, and evaluated *SPRY4* as a potential mediator of DT efficacy within the ATC–TAM axis.

## 2. Results

### 2.1. Dabrafenib and Trametinib IC_50_ in Anaplastic Thyroid Cancer Cells

To evaluate the intrinsic sensitivity of the *BRAF*-mutant (p.V600E) T235 and T238 ATC cells to DT, we examined the half maximal inhibitory concentration (IC_50_) for Dabrafenib and Trametinib monotherapies. The IC_50_ values and molecular features of each cell line are described in Table S1 and Figure S1. Based on the IC_50_ data, subsequent assays were performed using 10 nM of Dabrafenib plus 5 nM of Trametinib, as these concentrations in monotherapy led to 30–40% decrease in cell lines’ viability, thereby enabling the assessment of macrophage-mediated modulation of the response to DT combination, while avoiding excessive drug-induced cytotoxicity.

### 2.2. Macrophages Counteract the Anti-Tumour Effects of Dabrafenib Plus Trametinib Promoting an Increase in ATC Cell Viability

**Figure 1:**
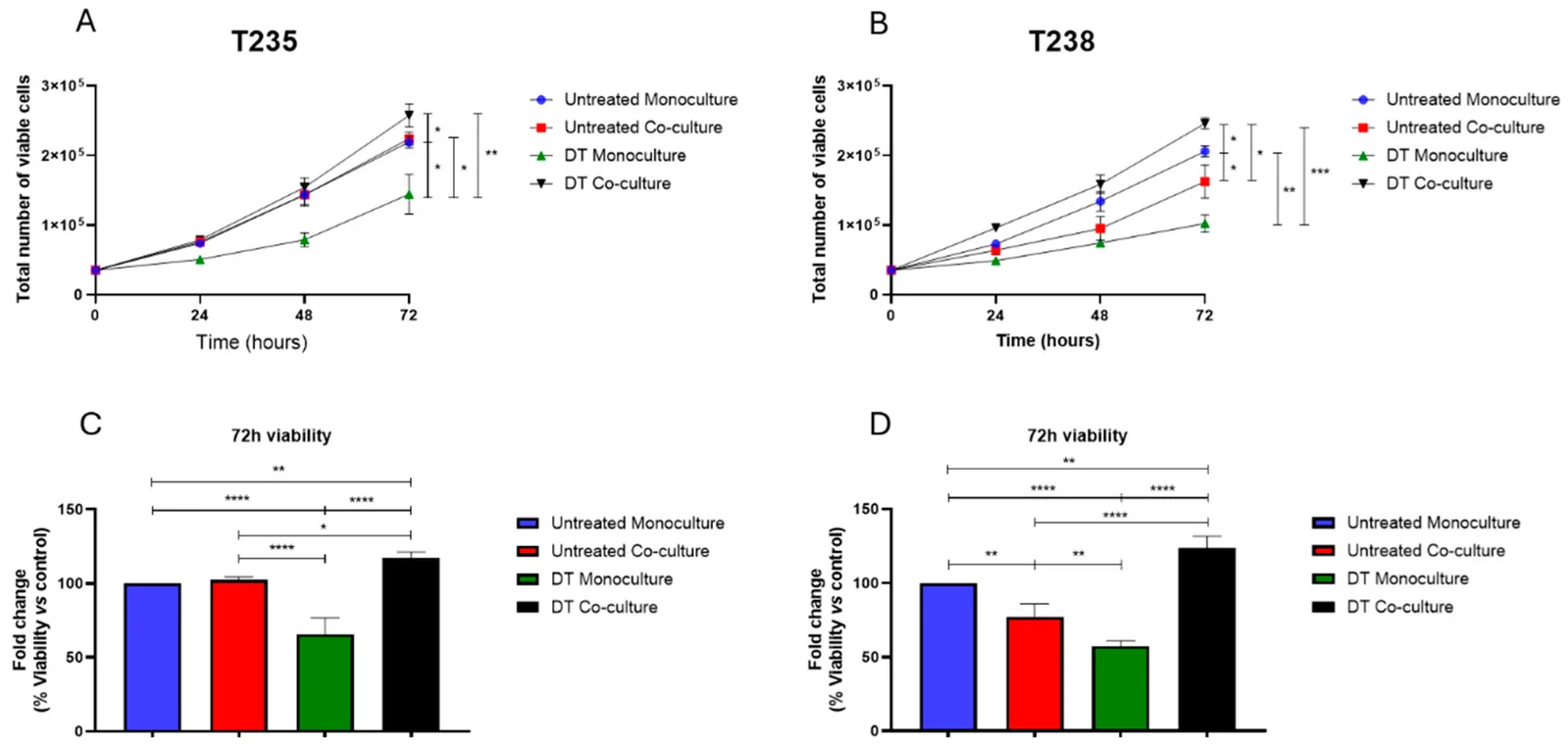
Cell viability of ATC cells in mono- and co-cultures, with or without Dabrafenib plus Trametinib. Viability assays of (**A**) T235 and (**B**) T238 for total number of cells throughout time and (**C**,**D**) for the % of viability at 72 h. The normalisation control (untreated monoculture) was defined as equal to 100. The values are represented as mean (standard deviation) from four independent biological assays, each with three technical replicates, and analysed using one/two-way ANOVA. * *p* < 0.05, ** *p* < 0.01, *** *p* < 0.001, **** *p* < 0.0001. Plots: blue—untreated monoculture, red—untreated co-culture, green—DT monoculture, black—DT co-culture.

To investigate the effects of macrophages in the efficacy of DT, we performed assays with mono- and co-cultures, with or without DT, and evaluated cell viability at 24, 48 and 72 h. The most marked responses were detected at 72 h of treatment (Figure 1; Table S2). As previously observed, in T235, macrophages (untreated co-culture) did not affect the intrinsic cellular viability [22] (Figure 1A), whereas in T238 macrophages induced a significant decrease in cellular viability (78.7%; *p* < 0.01) (Figure 1B), suggesting that macrophages differentially modulate the cellular viability depending on the cell line [22]. Upon DT treatment (DT monoculture), viability decreased in both cell lines (T235: 65.6% of viability, T238: 49.8% of viability; *p* < 0.0001 vs. untreated monoculture) (Figure 1). Notably, the addition of macrophages under DT treatment conditions (DT co-culture) resulted in increased viability in both cell lines, to levels exceeding those observed in the untreated monocultures (T235: 117.3%, T238: 119.7%; *p* < 0.0001 vs. untreated monoculture), suggesting that macrophages counteracted the anti-tumour effects of DT (Figure 1C,D).

Overall, DT treatment consistently reduced ATC cell viability. However, co-culture with macrophages significantly restored cell viability under DT treatment, suggesting that macrophages counteracted the anti-tumour effects of DT.

### 2.3. Macrophages Sustain the Invasive Phenotype of ATC Cells During DT Exposure

**Figure 2:**
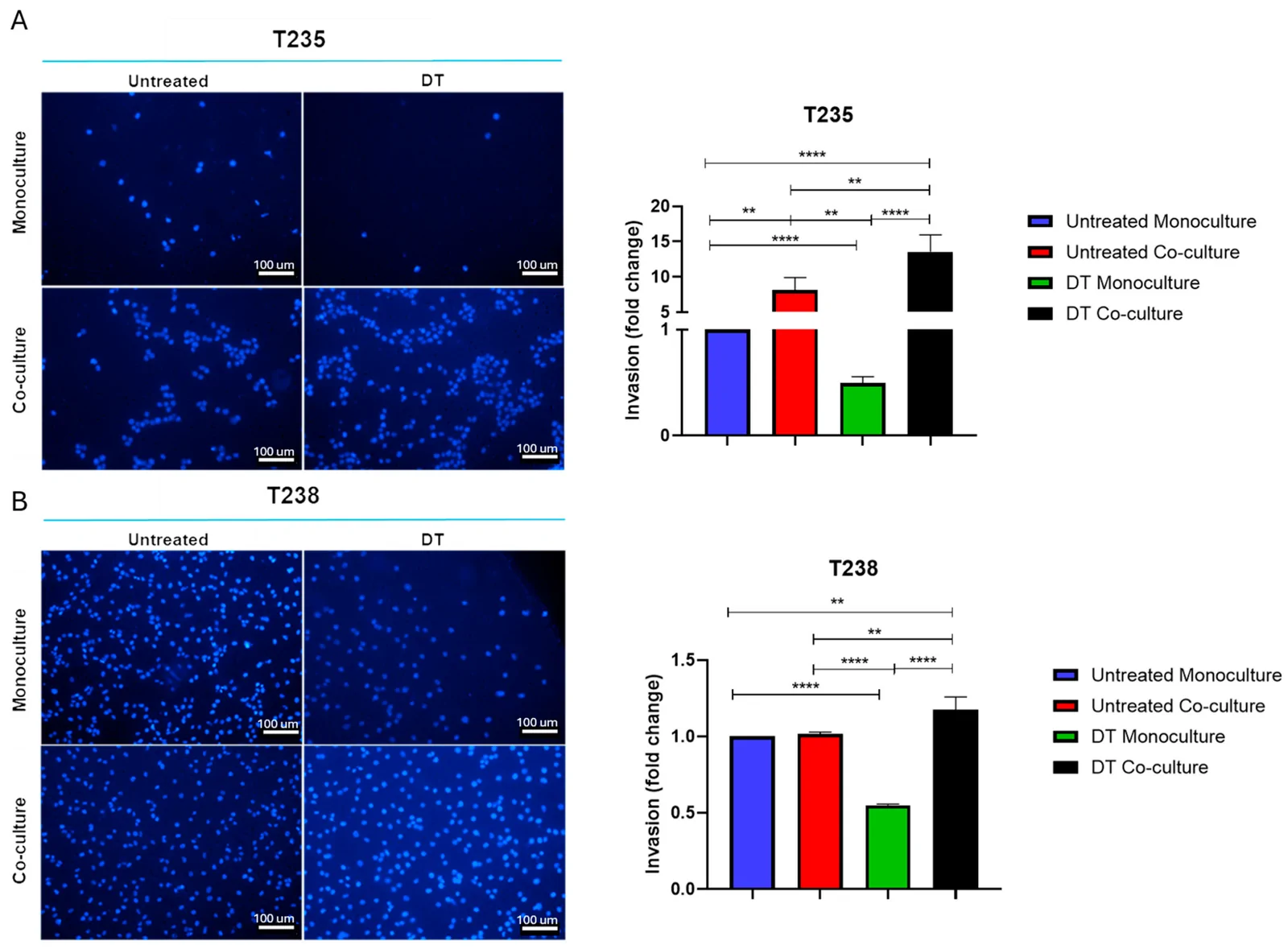
Invasive capability of the ATC cells in mono- and co-cultures, with or without Dabrafenib plus Trametinib. Representative images of the invasive cells for (**A**) T235 and (**B**) T238. The invasive cells were stained with DAPI (blue), counted and quantified, as presented in the graphs. The normalisation control (untreated monoculture) was defined as equal to 1. The values are represented as mean (standard deviation) from three independent biological assays, each with two technical replicates, and analysed using one-way ANOVA. Image scales at 100 μm at 10× magnification. ** *p* < 0.01, **** *p* < 0.0001. Plots: blue—untreated monoculture, red—untreated co-culture, green—DT monoculture, black—DT co-culture.

To evaluate the effects of DT and macrophages on the invasion capacity of the ATC cells, invasion assays were performed. As previously observed [22], macrophages (untreated co-culture) significantly incremented the invasion of T235 cells (*p* < 0.01 vs. untreated monoculture) (Figure 2A), whereas no changes were observed in the invasion capacity of T238 cells (Figure 2B), compared with untreated monoculture. Upon DT treatment (DT monoculture), invasion capacity significantly decreased in both ATC cell lines (*p* < 0.01; *p* < 0.0001 vs. untreated monoculture) (Figure 2). However, this effect was significantly attenuated when macrophages were introduced in the DT treatment conditions (DT co-culture), resulting in a significant increase in invasive capability, compared with the respective untreated monocultures (*p* < 0.01; *p* < 0.0001) (Figure 2).

Overall, these findings demonstrate that, while DT alone significantly reduces cell invasion, macrophages reverse this effect, increasing the invasive potential in both ATC cell lines.

### 2.4. Macrophages Induce Actin Cytoskeleton Remodelling and Increase Vimentin Expression in ATC Cells Under DT

Considering that cancer cell mobility and expression of epithelial-to-mesenchymal transition (EMT)-related genes are associated with invasion capability, we qualitatively evaluated the actin cytoskeleton and vimentin expression. We observed that T235 cells (Figure S2A), upon untreated co-culture, acquired a different actin cytoskeleton morphology, characterised by the presence of cellular protrusions, contrasting with their round shape in untreated monoculture, as previously reported for T235 [22]. The T238 cell line maintained the extensive morphology in the presence of macrophages (Figure S2B), as previously seen [22]. When DT was introduced (DT monoculture), T238 cells acquired a rounder shape (Figure S2B) and T235 maintained their round shape (Figure S2A). When macrophages were introduced under DT, both cell lines acquired a more elongated morphology. Similarly, vimentin expression changed in concordance with the presence of the actin protrusions and the invasion capability of the cells (Figure S2).

Overall, these results show that macrophages promote alterations in cellular morphology and vimentin expression in ATC cells, which are associated with increased motility and invasion. DT treatment made all cells rounder and likely less mobile, whereas macrophages reversed this effect. These changes in cell structure and vimentin expression are consistent with the increased invasion observed under co-culture conditions.

### 2.5. SPRY4 Expression Is Differentially Modulated in ATC Cells in Response to DT Therapy Under Mono- and Co-Culture Conditions

**Figure 3:**
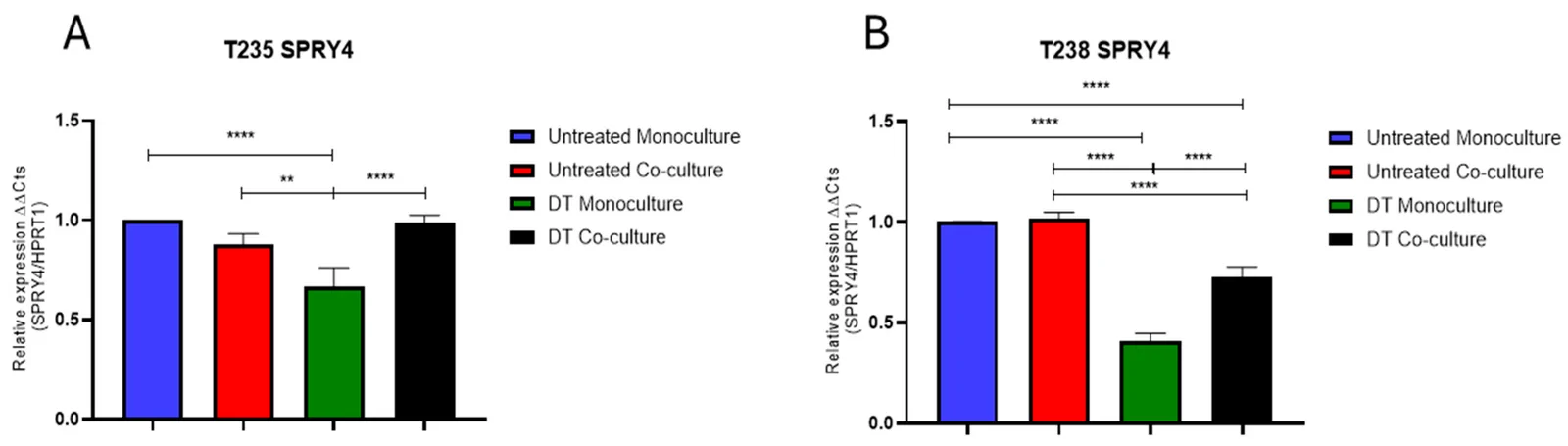
*SPRY4* gene expression in the two ATC cell lines in mono- and co-cultures, with or without Dabrafenib plus Trametinib. *SPRY4* expression levels in (**A**) T235 and (**B**) T238 cells. The normalisation control (untreated monoculture) was defined as equal to 1. The values are represented as mean (standard deviation) from four independent biological assays, each with three technical replicates, and analysed using one-way ANOVA. ** *p* < 0.01, **** *p* < 0.0001. Plots: blue—untreated monoculture, red—untreated co-culture, green—DT monoculture, black—DT co-culture.

To further understand the impact of DT and macrophages in the expression of *SPRY4* gene in ATC cells, we conducted qRT-PCR in mono- and co-cultures, with or without DT. In T235 untreated co-cultures, *SPRY4* expression slightly decreased (Figure 3A). On the other hand, in T238 untreated co-cultures, *SPRY4* maintained its intrinsic expression (Figure 3B). Notably, DT significantly reduced *SPRY4* in both cell lines (*p* < 0.01 vs. untreated monoculture) (Figure 3), but macrophage co-culture restored its expression, reaching control (untreated monoculture) levels in T235 and slightly below in T238 (*p* < 0.0001 vs. DT monoculture). In general, these results show that *SPRY4* expression is downregulated by DT treatment, while the presence of macrophages in co-culture totally or partially restores this expression. These findings raise the possibility that *SPRY4* may reflect ATC cell response to DT and possibly act as mediator, at least in part, of the modulatory effects exerted by macrophages.

### 2.6. MAPK and SPRY4 Protein Expression Are Differentially Modulated in ATC Cells in Response to DT Therapy Under Mono- and Co-Culture Conditions

To further investigate the effects of DT and macrophages on MAPK signalling and SPRY4 protein expression in ATC cells, we conducted Western blot analyses of SPRY4 and (p)ERK1/2 expression, in mono- and co-cultures, with or without DT.

**Figure 4:**
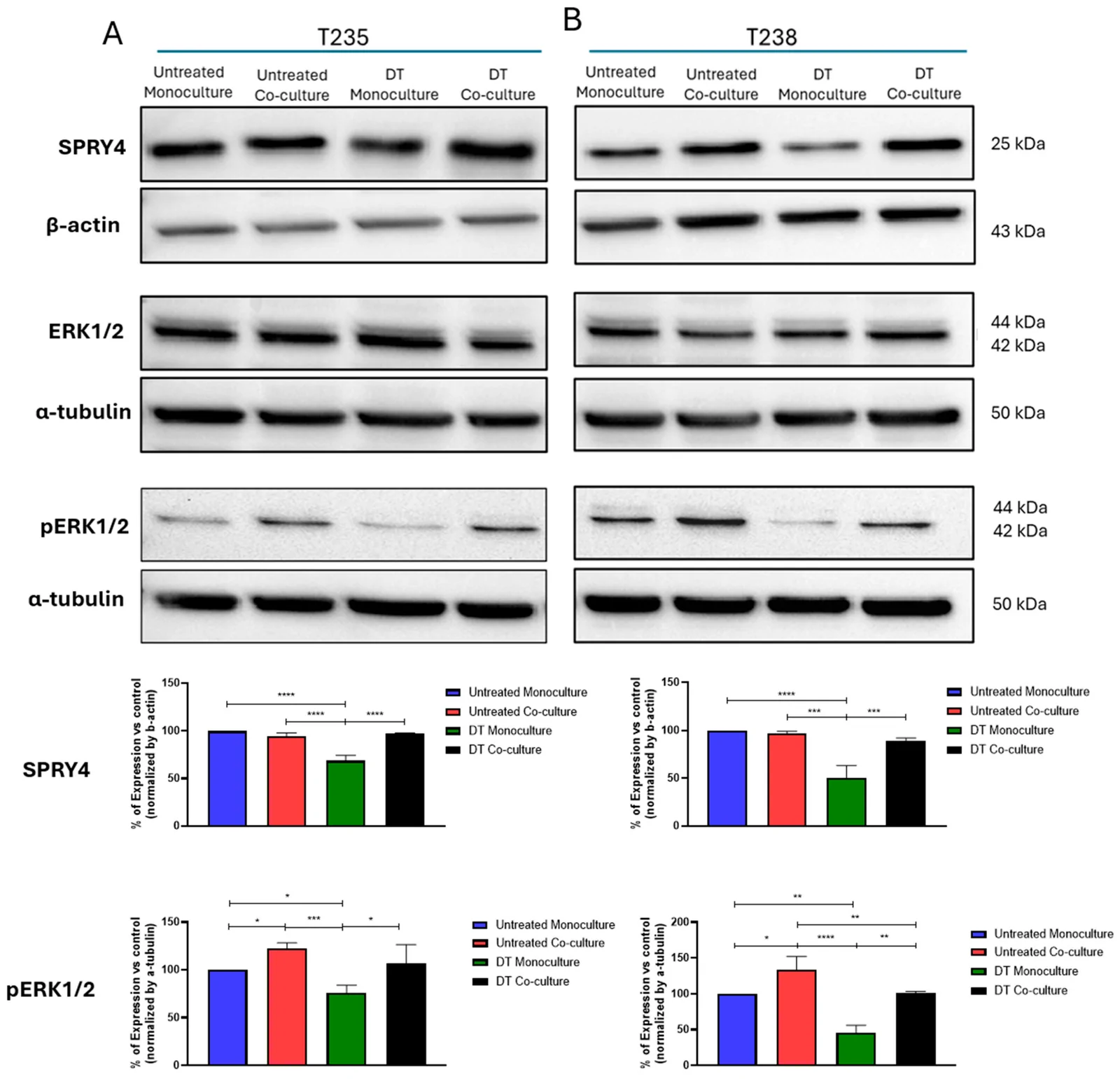
Western blot analysis of SPRY4 and (p)ERK1/2 in the ATC cell lines in mono- and co-cultures, with or without Dabrafenib plus Trametinib. Representative immunoblots for SPRY4, ERK1/2 and pERK1/2 for (**A**) T235 and (**B**) T238 cells, and graphical quantification for SPRY4 and pERK1/2. The normalisation control (untreated monoculture) was defined as equal to 100. The values are represented as mean (standard deviation) from three independent biological assays, each with two technical replicates, and analysed using one-way ANOVA. * *p* < 0.05; ** *p* < 0.01; *** *p* < 0.001; **** *p* < 0.0001. Plots: blue—untreated monoculture, red—untreated co-culture, green—DT monoculture, black—DT co-culture.

SPRY4 protein expression corroborated the gene expression results (Figure 4). No significant changes in SPRY4 protein levels were observed in either cell line in co-culture with macrophages, compared with the untreated monoculture, although a slight decrease was detected in T235 cells (Figure 4). Furthermore, DT treatment alone (DT monoculture) significantly decreased SPRY4 expression in both cell lines (*p* < 0.001; *p* < 0.0001 vs. untreated monoculture). However, in the presence of macrophages, concomitance with DT increased SPRY4 expression (*p* < 0.001; *p* < 0.0001 vs. DT monoculture), restoring expression to levels comparable to those observed in the untreated monoculture.

Regarding MAPK/ERK signalling, pERK1/2 levels were significantly increased in both cell lines under the untreated co-culture condition, compared with the control monoculture (*p* < 0.05; *p* < 0.01) (Figure 4). Upon DT treatment, in the absence of macrophages, pERK1/2 expression significantly decreased in both cell lines (*p* < 0.05; *p* < 0.01 vs. untreated monoculture), consistent with DT-mediated inhibition of the MAPK pathway. Interestingly, macrophage co-culture in the DT condition (DT co-culture) restored pERK1/2 expression compared with the DT monoculture (*p* < 0.05; *p* < 0.01), reaching levels similar to the untreated monoculture.

These findings suggest that macrophages modulate MAPK pathway activity in the presence of DT, and that SPRY4 expression follows the same pattern as MAPK/ERK signalling, supporting a potential relationship between the two. The full Western blot membranes are provided in Figure S3.

### 2.7. Co-Culture and DT Therapy Influence Macrophage Phenotype and PD-L1 Levels in ATC Cells

**Figure 5:**
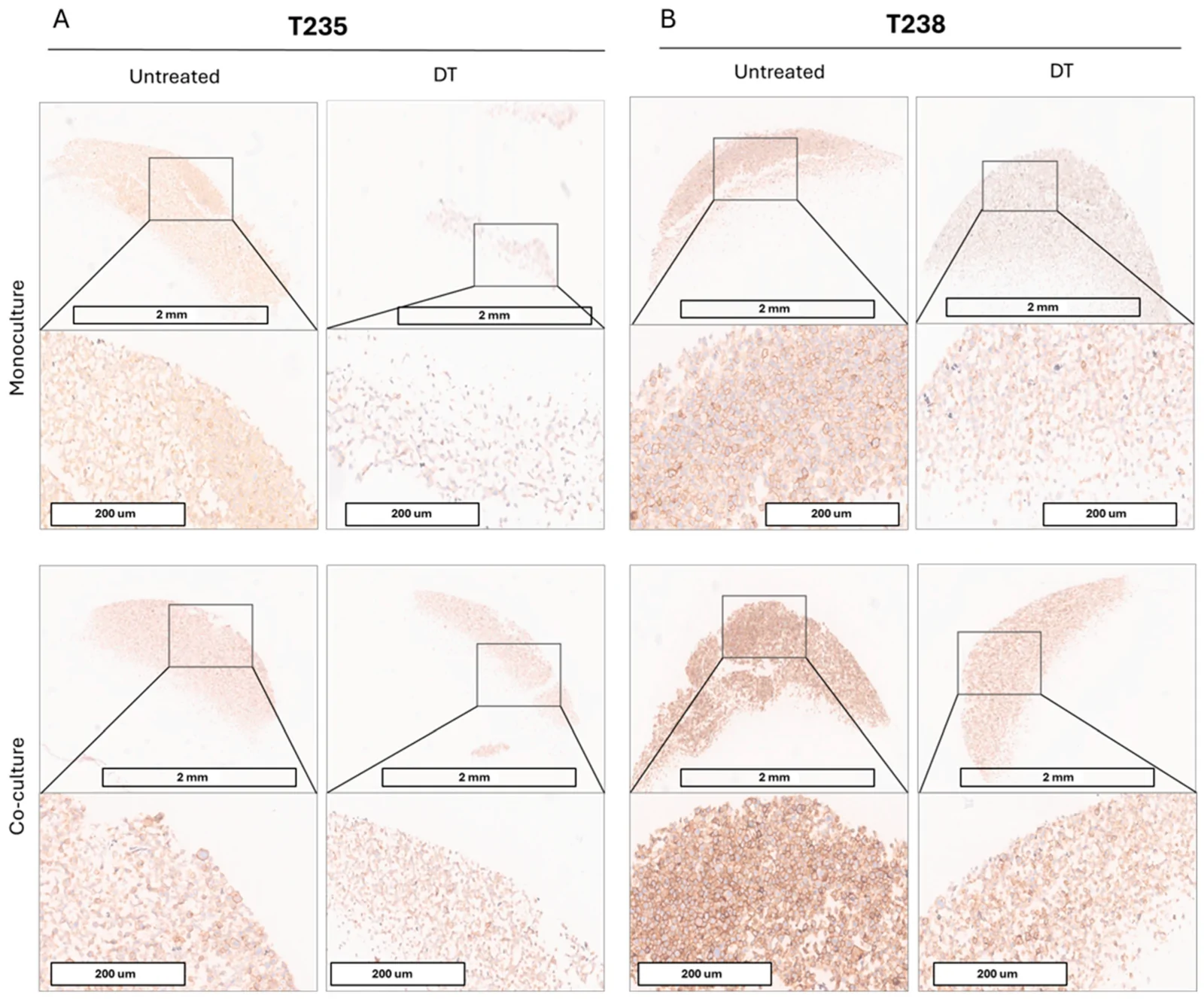
PD-L1 expression in ATC cell lines in mono- and co-cultures, with or without Dabrafenib plus Trametinib. Representative images of PD-L1 expression for (**A**) T235 and (**B**) T238 cells in monoculture and co-culture with and without DT, from three independent biological assays, each with two technical replicates. Original images are shown at a scale of 2 mm, with higher-magnification views at a scale of 200 μm.

To study the effects of DT treatment on PD-L1 expression in ATC cells, we conducted an immunohistochemistry analysis. We observed an increased PD-L1 expression in both co-culture conditions, untreated and with DT-treated, for both ATC cell lines (Figure 5) when compared with the untreated monocultures. Interestingly, DT treatment in monoculture (*i.e.,* in the absence of macrophages) led to decreased PD-L1 expression in both ATC cells, relative to the untreated monoculture condition (Figure 5; Table S3). These results suggested that both DT and macrophage interactions influence PD-L1 expression in ATC, and that macrophages are able to restore PD-L1 expression under DT treatment conditions.

**Figure 6:**
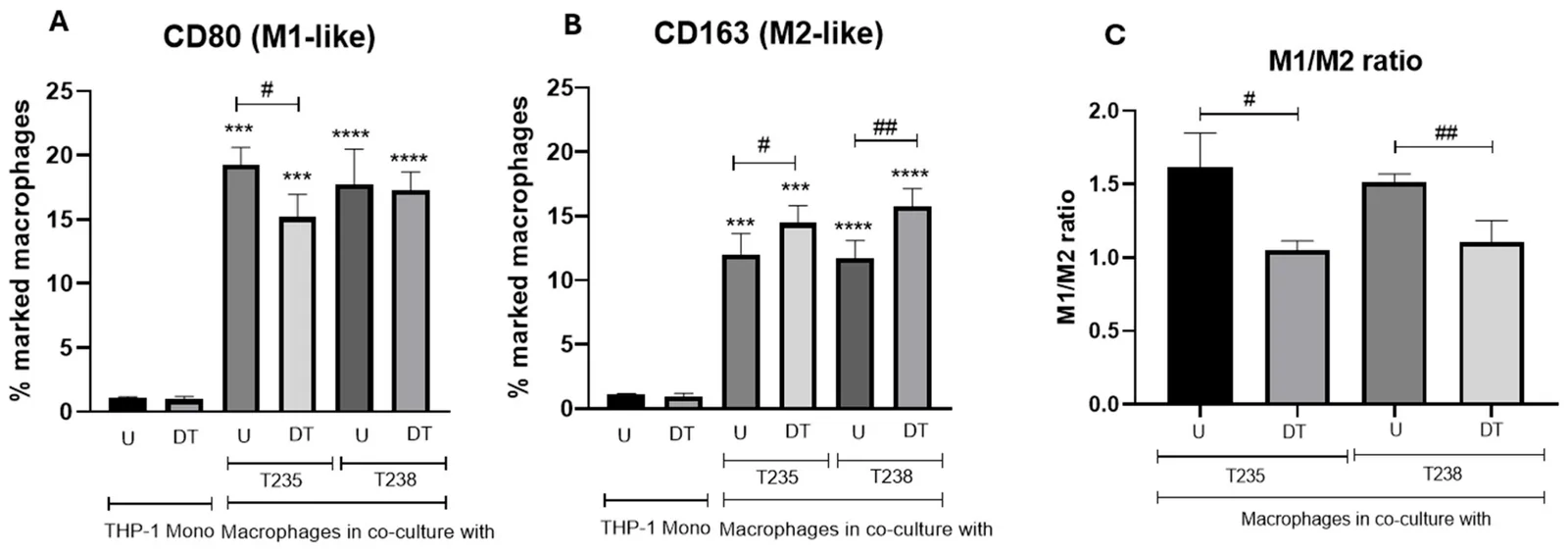
Flow cytometry analysis of CD80 and CD163 in THP-1-derived macrophages in mono- and co-cultures, with or without Dabrafenib plus Trametinib. (**A**) CD80 and (**B**) CD163 expression in THP-1-derived macrophages in co-culture with T235 and T238 cells, with or without DT therapy. (**C**) M1/M2 ratio of the THP-1-derived macrophages in co-culture with T235 and T238, with or without DT therapy. The main control (THP-1-mono U) was defined as equal to 1. The data was analysed from four independent biological assays, each with three technical replicates, using one-way ANOVA or unpaired parametric *t* test. A minimum of 10,000 total events were acquired for each sample. # *p* < 0.05, ## *p* < 0.01, *** *p* < 0.001, **** *p* < 0.0001. * represents the comparison between the conditions and the control THP-1-mono U. # represents the comparison between the conditions U and DT for each cell line. Mono—monoculture, Co—co-culture, U—untreated, DT—Dabrafenib plus Trametinib.

To investigate the effects on THP-1-derived macrophages polarisation, we analysed CD80 and CD163 by flow cytometry. Macrophages expressed CD80 and CD163 in all experimental conditions. For CD80 expression (Figure 6A), both untreated and DT-treated conditions showed significantly higher expression, compared with the control (THP-1-derived macrophages monoculture) (*p* < 0.001, *p* < 0.0001). However, a significant decrease in CD80 expression was observed in THP-1-derived macrophages co-cultured with T235 under DT, compared with the corresponding untreated condition (*p* < 0.05). Regarding CD163 expression (Figure 6B), similar trends were observed in both cell lines when compared with the control. In addition, DT treatment significantly increased CD163 expression in both cell lines, compared with the corresponding untreated conditions (*p* < 0.05). Furthermore, the M1/M2 ratio was lower in DT-treated cells than in untreated cells, suggesting a shift towards a more pro-tumoural macrophage phenotype.

These results suggest that DT treatment influences macrophage polarisation by promoting a transition towards intermediate M1/M2 or markedly M2-like phenotype.

### 2.8. Targeting Macrophages with Edicotinib Reduces Macrophage-Mediated Pro-Tumoural Effects and Restores DT-Induced Inhibition on ATC Cell Viability

**Figure 7:**
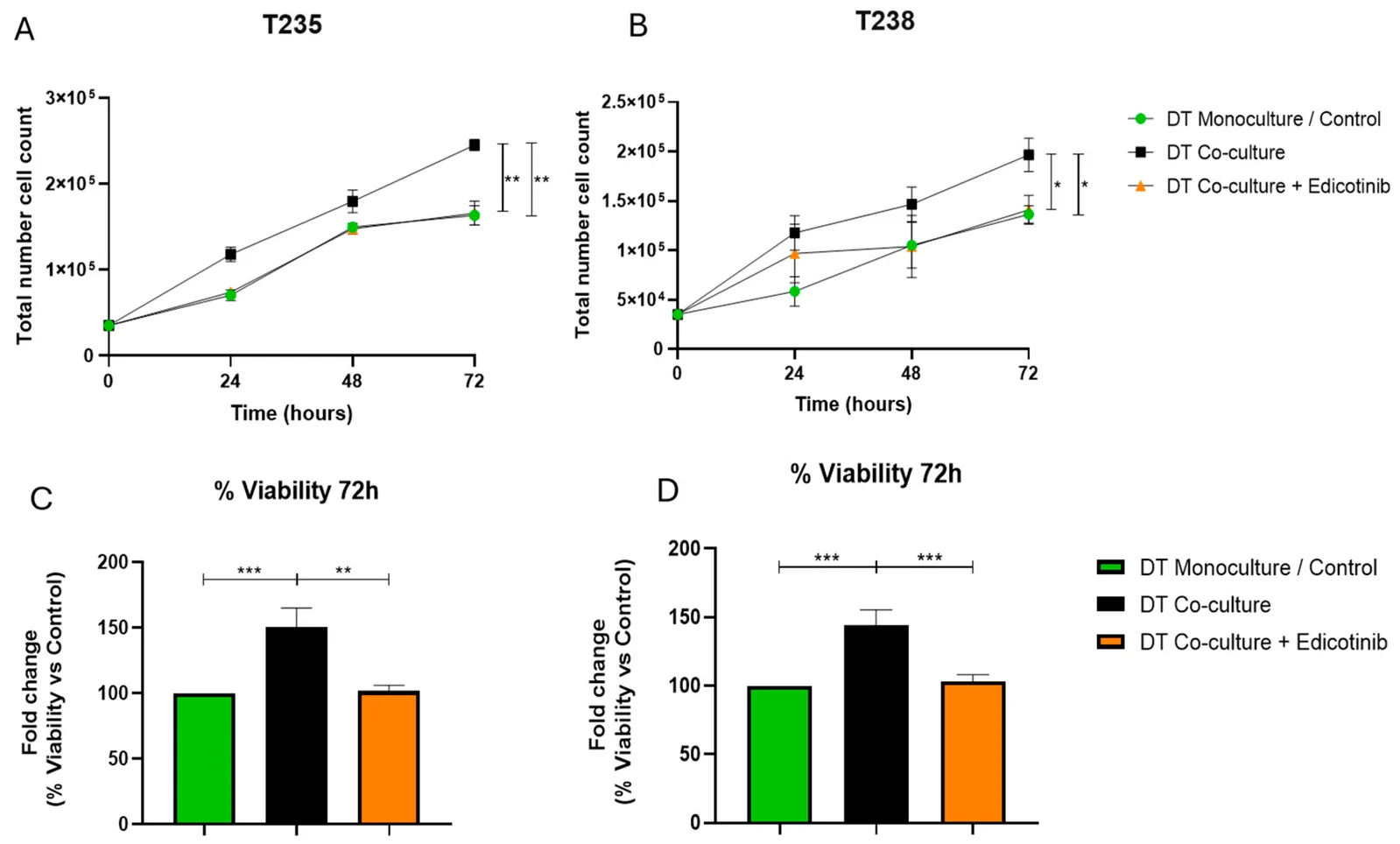
Cell viability of T235 and T238 in mono- and co-cultures with DT and with or without Edicotinib. Viability assays of (**A**) T235 and (**B**) T238 for total number of cells throughout time and (**C**,**D**) for the % of viability at 72 h. The normalisation control (DT monoculture) was defined as equal to 100. The values are represented as mean (standard deviation) from three independent biological assays, each with two technical replicates, and analysed using one/two-way ANOVA. * *p* < 0.05, ** *p* < 0.01, *** *p* < 0.001. Plots: green—DT monoculture, black—DT co-culture, orange—DT co-culture with Edicotinib.

Considering the role of macrophages in modulating DT efficacy in ATC cells, we investigated whether macrophage targeting with Edicotinib, a CSF1R inhibitor [23], could restore or enhance the effects of DT treatment. As such, viability assays were conducted overtime, as described above, in mono- and co-cultures with DT, in the presence or absence of Edicotinib. As observed previously, the most pronounced response of ATC cells in monoculture was detected after 72 h of treatment (Table S4). Pharmacological inhibition of macrophages with Edicotinib in combination with DT therapy significantly reduced the total number of cells overtime in both ATC cell lines (*p* < 0.05) (Figure 7A,B), and the viability of T235 (Figure 7C) and T238 (Figure 7D) cells, at 72 h, by approximately 50% (*p* < 0.01) and 40% (*p* < 0.001), respectively, restoring viability to levels comparable to those observed in DT-treated monocultures. We also observed that Edicotinib does not appear to have an additive effect on ATC cells under DT therapy, which supports that macrophages are the primary target of Edicotinib (Figure S4).

These results highlight macrophage targeting as a promising strategy to enhance the therapeutic response to DT in ATC.

### 2.9. Targeting Macrophages with Edicotinib Blocked the Inhibitory Effects of Macrophages in DT Therapy in ATC Cells Invasion

**Figure 8:**
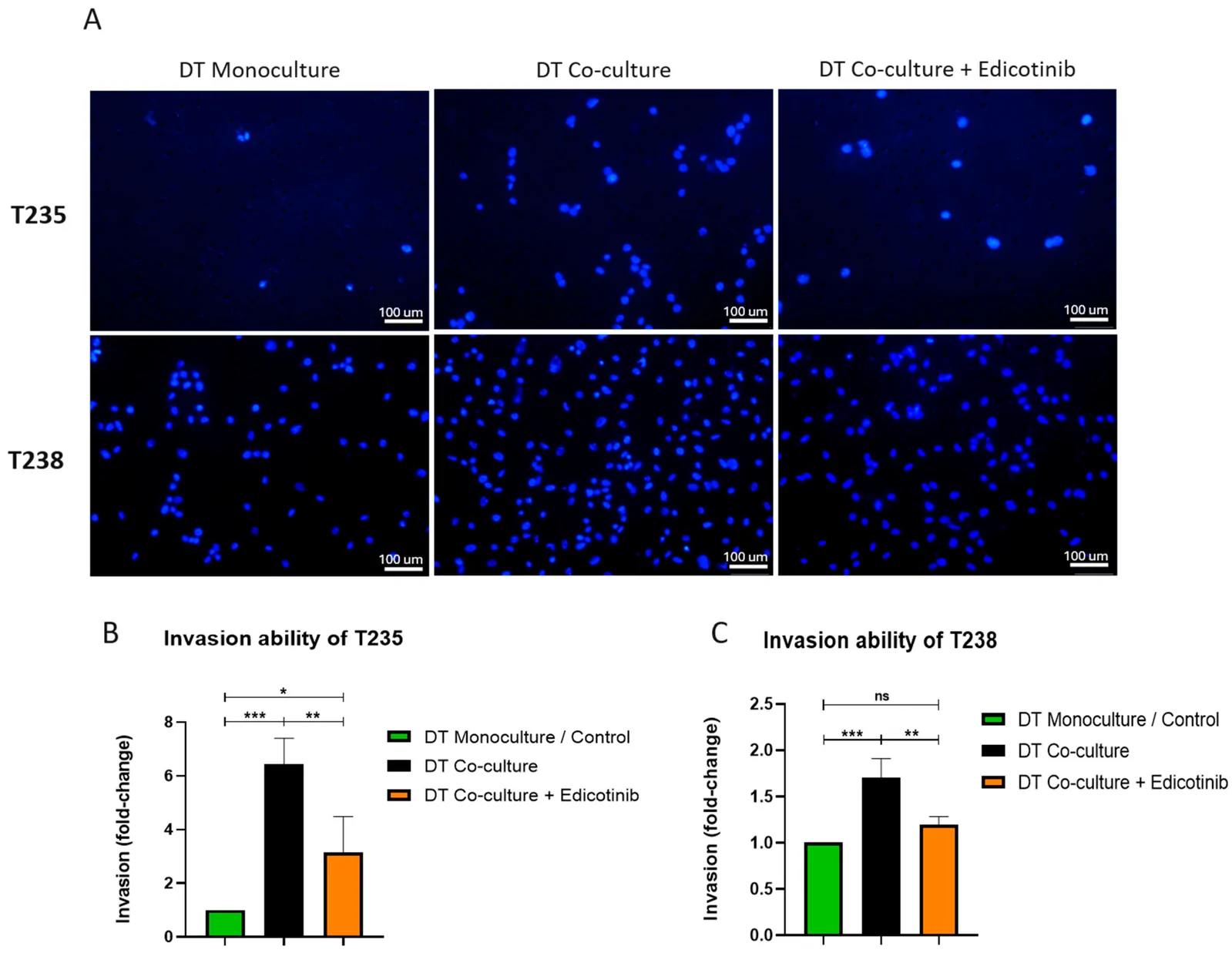
Invasion ability of T235 and T238, in mono- and co-cultures with Dabrafenib plus Trametinib and with or without Edicotinib. Representative images of the invasive cells and graphical representation for (**A**,**B**) T235 and (**A**,**C**) T238. The invasive cells were stained with DAPI (blue), counted and quantified as presented in the graphs. The normalisation control (DT monoculture) was defined as equal to 1. The values are represented as mean (standard deviation) from three independent biological assays, each with two technical replicates, and analysed using one-way ANOVA. Image scales at 100 μm at 20× magnification. ns, non-significant, * *p* < 0.05, ** *p* < 0.01, *** *p* < 0.001. Plots: green—DT monoculture, black—DT co-culture, orange—DT co-culture with Edicotinib.

Building on the observed macrophage-mediated resistance to DT, we investigated whether pharmacological inhibition of macrophages could restore the anti-invasive effects of DT in ATC cells. Macrophage co-culture under DT increased invasion by approximately 6.5-fold and 1.5-fold, in T235 and T238, respectively, compared with DT-treated monocultures (*p* < 0.001) (Figure 8A–C), consistent with previous observations. However, in T235 cells the addition of Edicotinib reduced invasion to approximately 3-fold relative to DT monoculture conditions (*p* < 0.05) (Figure 8A,B), indicating a partial reversal of macrophage-mediated pro-invasive effects. In T238 cells Edicotinib almost completely reversed the macrophage-induced pro-invasive response, reducing the invasion to levels comparable to DT-treated monocultures (Figure 8A,C).

Collectively, these findings demonstrate that Edicotinib effectively counteracts macrophage-driven pro-invasive effects in ATC cells under DT treatment, highlighting its potential to modulate macrophage-mediated resistance mechanisms and to enhance DT efficacy.

### 2.10. Edicotinib Reduces M2-like Macrophage Polarisation and Shifts the M1/M2 Balance Toward a Less Predominant Pro-Tumoural Phenotype

**Figure 9:**
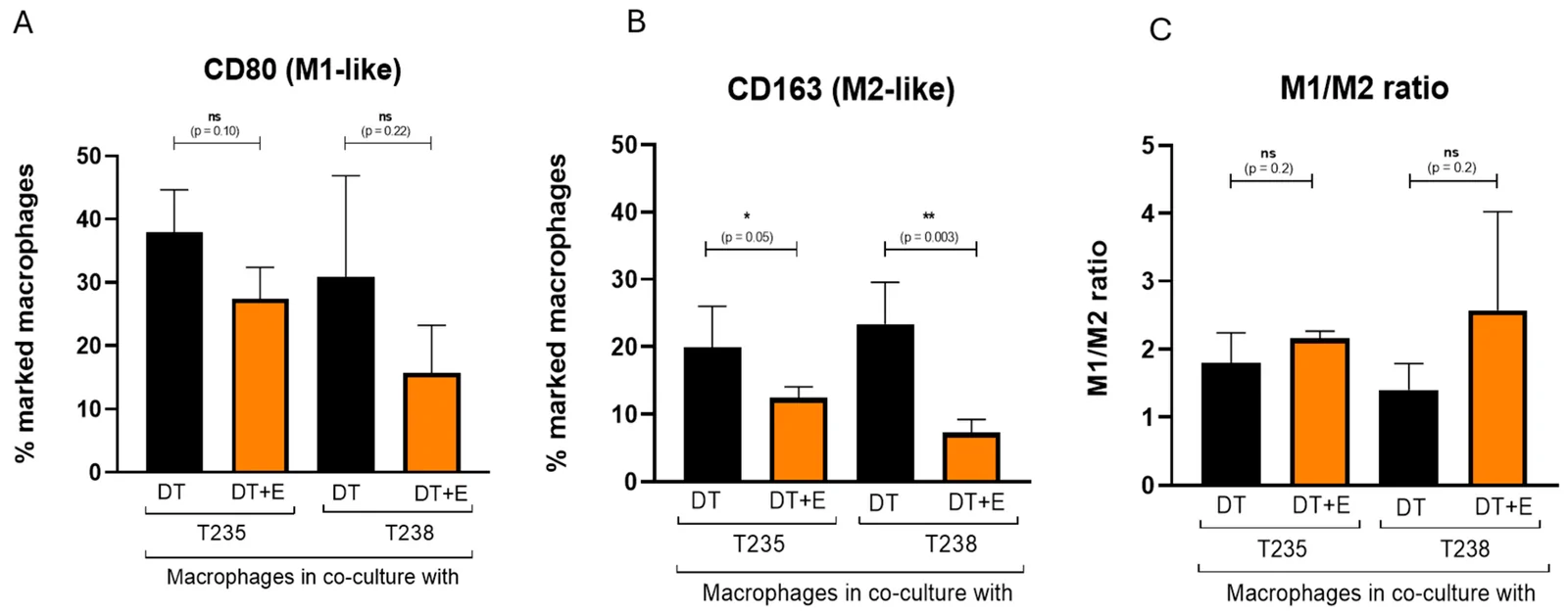
Flow cytometry analysis of CD80 and CD163 in THP-1-derived macrophages in mono- and co-cultures, with Dabrafenib plus Trametinib, and with or without Edicotinib. (**A**) CD80 (M1-like) and (**B**) CD163 (M2-like) expression in THP-1-derived macrophages, in co-culture with T235 and T238 cells, under DT therapy in the presence or absence of Edicotinib. (**C**) M1/M2 (CD80^+^/CD163^+^) ratio of the THP-1-derived macrophages. The values are represented as mean (standard deviation) from three independent biological assays, each with three technical replicates, and analysed using the unpaired parametric *t* test. A minimum of 10,000 total events were acquired for each sample. ns, non-significant, * *p* < 0.05, ** *p* < 0.01. black—DT co-culture, orange—DT co-culture with Edicotinib. DT, Dabrafenib plus Trametinib; DT + E, Dabrafenib plus Trametinib combined with Edicotinib.

To evaluate the effects of Edicotinib on macrophage polarisation, CD80 and CD163 expressions were analysed by flow cytometry in THP-1-derived macrophages in co-culture with ATC cells under DT. Edicotinib did not significantly affect the proportion of CD80^+^ (M1-like) macrophages, although a modest decrease was observed, particularly in T238 co-cultures treated with DT plus Edicotinib, compared with DT alone (Figure 9A). In contrast, Edicotinib significantly reduced the percentage of CD163^+^ (M2-like) macrophages in co-culture with both ATC cell lines treated with DT plus Edicotinib (T235: *p* < 0.05; T238: *p* < 0.01) (Figure 9B). Consistent with these findings, treatment with Edicotinib resulted in a trend towards an increased M1/M2 ratio (CD80^+^/CD163^+^) in both co-culture models (Figure 9C).

Collectively, these results indicate that Edicotinib markedly reduces the M2-like macrophage population in DT-treated co-cultures, while exerting only minimal effects on the M1-like population. This shift towards a higher M1/M2 ratio suggests that Edicotinib may promote a less predominant pro-tumoural macrophage phenotype.

### 2.11. BRAF p.V600E ATC Tumours Exhibit Extensive Macrophage Infiltration with Predominant M2-like Polarisation

**Figure 10:**
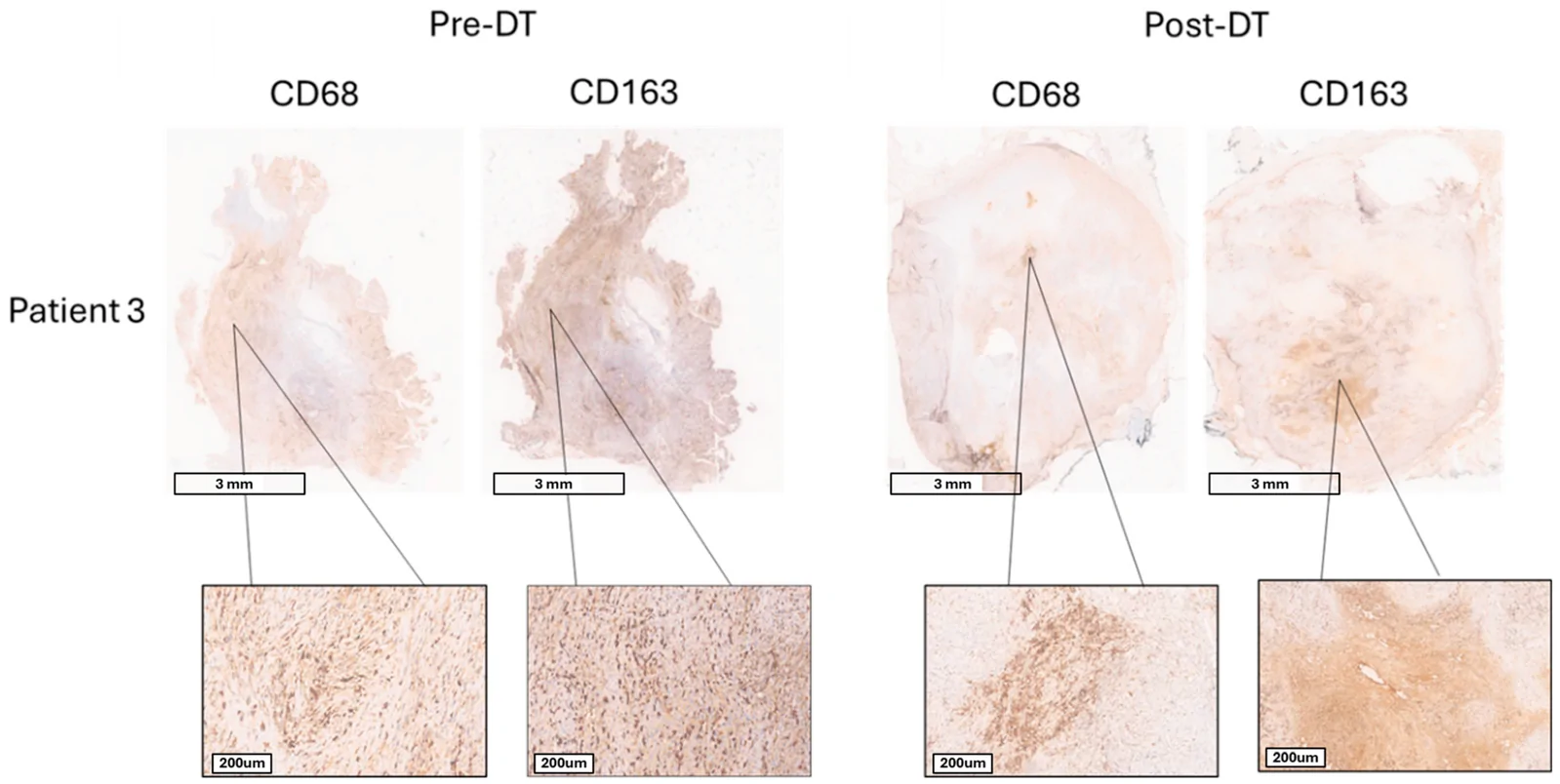
Macrophage infiltration in *BRAF*-mutant ATC samples of patients under DT. Representative pictures of CD68 and CD163 expression in ATC tumour samples from one patient, before and after the start of DT treatment. Original images are shown at a scale of 3 mm, with higher-magnification views at a scale of 200 μm.

To translate our *in vitro* findings to the clinical setting, we qualitatively analysed, by IHC, ATC tumour samples from eleven patients with *BRAF*-mutant ATC treated with DT, at baseline (pre-DT) and after treatment (post-DT). The tumours displayed extensive macrophage infiltration, as indicated by strong CD68 positivity, in both pre- and post-DT samples (Figure 10; Table S5). Notably, CD163 expression largely overlapped with CD68, indicating that the majority of tumour-infiltrating macrophages exhibited an M2-like phenotype. Representative analysis from one patient (Patient 3) is shown in Figure 10.

## 3. Discussion

Dabrafenib plus Trametinib (DT) therapy has become the standard treatment for patients with *BRAF* p.V600E-mutant ATC, and has significantly improved overall survival (OS) and progression-free survival (PFS) in these patients [6,7,8,9,10]. However, resistance can develop through (epi)genetic alterations, changes in gene expression, parallel pathway activation, and influences from the tumour microenvironment (TME). The ATC’s TME is highly enriched in macrophages, which account for approximately 50% of the tumour mass, forming a dense interconnected network that supports tumour growth and vascularisation [18,19,24]. Macrophage infiltration has been associated with poor prognosis, metastasis, extrathyroidal extension and aggressiveness, suggesting a critical role in TC progression [19]. Studies in melanoma and TC have also linked macrophages to resistance to BRAF inhibitors monotherapy, as these cells tend to acquire a more pro-tumoural phenotype [17,20,25].

Here, we investigated the influence of macrophages on the response to DT treatment in ATC. Consistent with our previous study on the role of macrophages in promoting the aggressiveness of ATC cells [22], we found that, although DT treatment reduced the viability of ATC cell lines, when these tumour cells were co-cultured with macrophages the reduction in viability was markedly attenuated and the ATC cell viability was restored to levels exceeding those observed in untreated monocultures. Similarly, the macrophage-mediated protection against targeted therapies and chemotherapy has also been reported in melanoma, lung cancer and other tumour models, in cell lines and in patients [25,26,27,28]. The present findings suggest that macrophages can reduce the anti-tumour effects of DT, possibly by contributing to a more pro-tumoural environment that supports ATC cell survival. In addition, we observed that macrophages reversed DT-induced suppression of invasion, promoted cytoskeletal reorganisation, increased cellular protrusions, and incremented vimentin expression. Together, these findings suggested that macrophages not only protect ATC cells from DT-induced loss of viability, but also sustain their invasive and EMT-like properties.

*SPRY4* has been implicated in cell proliferation, survival, differentiation and EMT, and, as previously shown by our group, is a likely mediator of the ATC–macrophage axis with a tumour suppressor role [21,22,29,30,31]. Based on this, we evaluated the influence of DT therapy in *SPRY4* expression, under ATC–macrophage co-culture. DT treatment alone consistently reduced *SPRY4* gene expression, whereas co-culture with macrophages under DT partially or fully restored *SPRY4* levels, depending on the ATC cell line. Protein analysis confirmed these findings and also showed a parallel increase in pERK1/2 in the macrophage plus DT condition. Thus, in the context of DT treatment, *SPRY4* expression appeared to be predominantly regulated by MAPK-dependent feedback. The concomitant recovery of SPRY4 expression and increased ERK1/2 activation suggest that macrophage-derived signals can counteract DT-mediated suppression of the MAPK pathway. Given that SPRY4 is itself a MAPK-responsive gene [30], its re-expression may reflect reactivation of the pathway and engagement of a feedback loop. Additional macrophage-related signalling pathways may also contribute to this response, highlighting the complexity of macrophage-mediated regulation during DT treatment.

Given that cancer cells and macrophages can mutually influence the immune checkpoint (IC) modulation, we analysed PD-L1 expression in ATC cells and further explored the effects of DT and macrophage in this context. DT treatment reduced PD-L1 expression in ATC cells, while this effect was reversed in the presence of macrophages, which restored PD-L1 levels under DT conditions. These alterations are consistent with evidence linking MAPK signalling to PD-L1 regulation. Indeed, in lung adenocarcinoma and melanoma cancer cells, MAPK inhibition reduced PD-L1 expression [32,33], while *BRAF* p.V600E mutations in papillary TC were associated with an increased PD-L1 expression [34], supporting a role for MAPK activation in upregulating PD-L1. Moreover, in ATC, higher expressions of PD-L1 and PD-1 have been associated with worse OS and PFS [35]. Therefore, the observed effects of DT and macrophages on PD-L1 expression provide mechanistic insights into IC modulation in ATC, with potential utility for the combination of DT with IC inhibitors in cases with DT resistance.

Macrophage polarisation is influenced by the interactions with the cancer cells and the TME, significantly influencing therapeutic outcomes. These immune cells exhibit remarkable plasticity and can dynamically transition between activation states in response to signals from the TME, resulting in a continuum of phenotypes (M1-like and M2-like) rather than the two extremes (M1 and M2) of this phenotypic spectrum [16,17,18,19]. In our cohort of patients with ATC, a qualitative IHC analysis of macrophage in tumour samples showed strong infiltration of M2-like macrophages, which was also observed by others [16,19,24], further supporting their role in ATC progression and aggressiveness [26,28]. In the context of DT treatment, our *in vitro* macrophage analyses showed that DT influenced macrophage polarisation towards an intermediate phenotype, with a more pro-tumoural behaviour (M2-like). In addition, in the IHC analyses of ATC from patients under DT treatment, we observed that most tumours had a high infiltration of M2-like macrophage populations, with predominant CD163 expression. Consistently, Lee et al. reported increased infiltration of activated M2-like macrophages in ATC and aggressive PTC, upon exposure to BRAF inhibitor (Vemurafenib) [20]. Furthermore, Angell et al. reported a trend towards a higher CD163 expression compared with CD80^+^ in patients with *BRAF*-mutant aggressive differentiated thyroid tumours, although with marked inter-patient variability [34].

Our observations underscore the bidirectional nature of ATC–macrophage interactions and the dynamic modulation of the immune microenvironment by DT therapy. Macrophages appear to counteract the inhibitory effects of DT, with their polarisation state playing a key role in this process. This suggests that macrophages may represent a promising target for immune-based therapies, including anti-CSFR and anti-PD-L1 approaches [17,26,36,37], that could be combined with DT. Edicotinib inhibits CSF1–CSF1R signalling by blocking CSF1R autophosphorylation and the downstream pathways, such as MAPK, PI3K/AKT/mTOR and, preferentially, the JAK/STAT in macrophages [38,39]. The normal CSF1-driven JAK2/STAT3 activation promotes macrophage survival, proliferation, and expression of immunosuppressive mediators, reinforcing a M2-like phenotype [39,40,41]. Furthermore, CSF1 is involved in the recruitment and survival of naïve macrophages within the TME [39,40,41,42]. We therefore hypothesised that targeting macrophages in combination with DT could restore its inhibitory effects. To test this hypothesis, DT was combined with Edicotinib in an *in vitro* ATC–macrophage co-culture model. Combined treatment markedly reduced macrophage-mediated viability and macrophage-driven invasion of ATC cells, restoring both to levels comparable to those observed in DT-treated monocultures. These results suggest that Edicotinib counteracts macrophage-mediated resistance and pro-invasive behaviour, enhancing DT efficacy. Furthermore, Edicotinib seems to promote a less predominant pro-tumoural macrophage phenotype by specifically targeting M2-like macrophages, highlighting its ability to modulate the TME and potentially enhance DT therapeutic responses in ATC.

**Figure 11:**
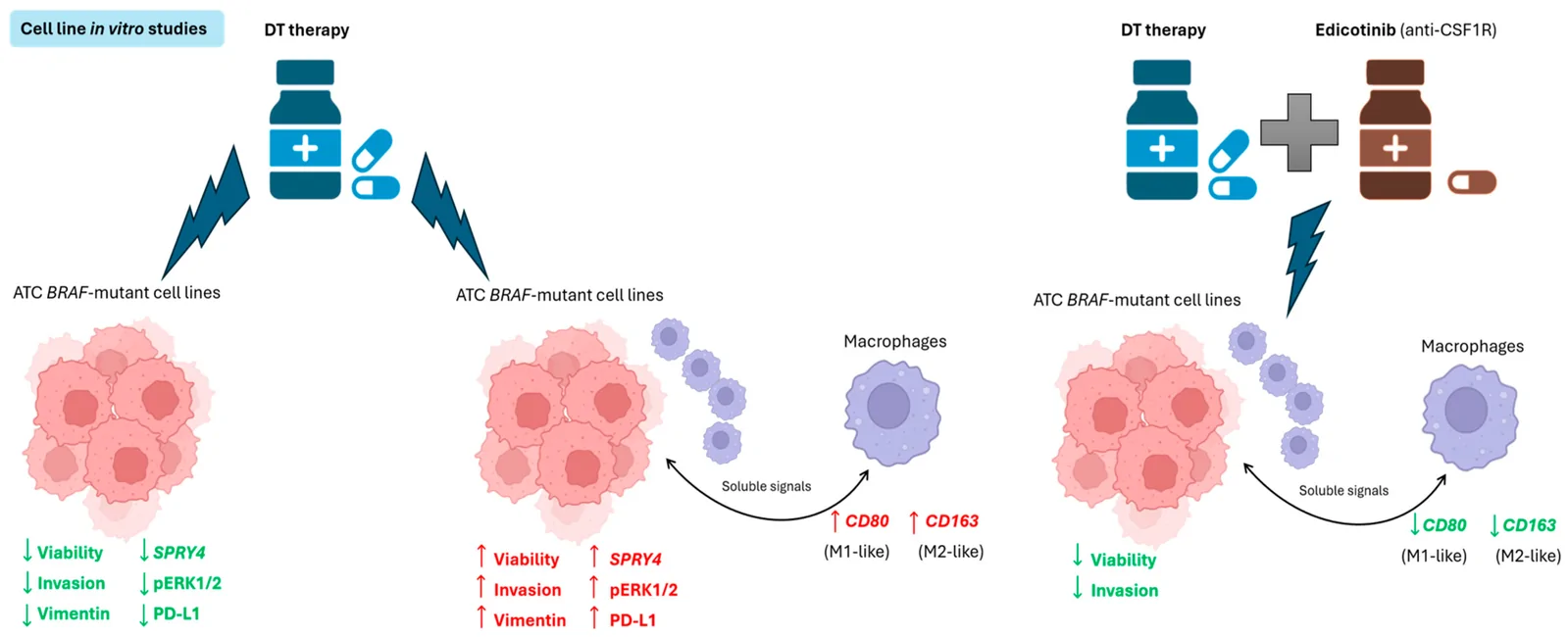
Macrophages as drivers of resistance to DT therapy in ATC. DT therapy induces the decrease in viability, invasion and related structures, vimentin expression, SPRY4, and PD-L1 by inhibiting the MAPK pathway. The introduction of macrophages in the system bypasses the inhibitory effect of DT and promotes an overexpression of CD80 and CD163 in macrophages. Furthermore, targeting macrophages with Edicotinib counteracted the macrophage-mediated DT resistance and decreased macrophage polarisation towards a less predominant M2-like phenotype. ↑, increased expression or function; ↓, decreased expression or function.

This study has several strengths, particularly its novelty as it is, to our knowledge, the first to investigate the role of macrophages in modulating DT efficacy in TC, building on our previous work [22]. We showed by *in vitro* studies that macrophages influence DT response by affecting ATC cell viability, invasion, MAPK signalling, and PD-L1 expression, potentially in association with their polarisation state. These findings were further reinforced by macrophage-targeting experiments, which supported the influence of macrophages in DT response, and unveiled a potential strategy to overcome macrophage-mediated DT resistance. Patient tissue analyses demonstrated substantial macrophage infiltration before and after treatment, supporting macrophages as potential therapeutic targets in DT-based combination strategies (Figure 11). This study included a relatively small number of ATC patients’ samples, reflecting the rarity of ATC (accounting for only 1–3% of all thyroid cancer cases), its highly aggressive clinical course, the limited number of patients eligible for treatment with DT (approximately 50% of ATC patients harbour the *BRAF* p.V600E mutation), and the unresectability of most of these tumours. Further investigation of macrophage-related features in larger ATC cohorts, together with their association with patients’ clinicopathological characteristics, is warranted to confirm the clinical relevance of our findings. Furthermore, cytokine and chemokine profiling, analysis of a broader panel of macrophage polarisation markers, and validation in *in vivo* models represent important directions for future studies that may provide further insight.

Overall, our findings support the therapeutic targeting of the CSF1/CSF1R signalling cascade within the ATC–macrophage axis as a promising target to improve DT therapeutic efficacy.

## 4. Materials and Methods

### 4.1. Patients

Biopsy or surgical samples from eleven ATC patients under DT, followed up at the Instituto Português de Oncologia de Lisboa Francisco Gentil (IPOLFG), were used in this study. IPOLFG is a referral centre for ATC for several hospitals, from the centre and south of Portugal to the islands (estimated total population of around 7 million people). This study was approved by the Ethical Committee of IPOLFG. Informed consent was obtained from all patients. This research was conducted in agreement with the World Medical Association Declaration of Helsinki.

### 4.2. Cell Lines and Culture

Two human *BRAF*-mutant (p.V600E) ATC cell lines (T235 and T238) and one monocytic leukaemia cell line (THP-1) were used in this study. The T235 and T238 ATC cell lines were selected as appropriate *in vitro* models for investigating the response to DT, due to the presence of the *BRAF* actionable mutation. The THP-1 cell line was selected as a well-established human monocytic model for macrophage differentiation and was used to investigate tumour–macrophage interactions in co-culture experiments [22]. These cell lines were also used in our previous study [22], enabling direct comparison with our earlier findings.

T235 and T238 were kindly supplied by Dr. Lúcia Roque (IPOLFG) and THP-1 was generously supplied by Dr. Raquel Gonçalves from i3S (Porto, Portugal). The cells were cultured in RPMI-1640 medium with HEPES (Gibco, Life Technologies, Paisley, UK) supplemented with 10% (*v*/*v*) foetal bovine serum (FBS) (Gibco), 1% (*v*/*v*) penicillin-streptomycin (Gibco), and 1% (*v*/*v*) L-glutamine (Gibco). All cells were incubated at 37 °C, in a humidified 5% CO_2_ incubator. Macrophages were generated by exposing the THP-1 to Phorbol 12-Myristate 13-Acetate (PMA; Sigma-Aldrich, St. Louis, MO, USA), as reported by Pinto et al. [22].

### 4.3. Therapeutic Drugs

Dabrafenib (cat. no. S2807, purity ≥ 99%), Trametinib (cat. no. S2673, purity ≥ 99%) and Edicotinib [cat. no. S8524, purity ≥ 99%; anti-colony stimulation factor 1 receptor (CSF1R)] were purchased from Selleck Chemicals (Houston, TX, USA) and dissolved in dimethylsulfoxide (DMSO; Honeywell, Charlotte, NC, USA). All drugs were reconstituted from lyophilised powder in 100% (*v/v*) DMSO, according to the manufacturers’ protocols and based on their solubility at 25°C: Dabrafenib and Trametinib stock solutions were prepared at 5 mM, while Edicotinib was prepared at 2 mM. Appropriate working solutions were subsequently prepared by dilution in DMSO. These were then added to the culture medium to achieve the desired final drug concentrations, with a final DMSO concentration of 0.5% (*v/v*). Dabrafenib and Trametinib were diluted in a medium supplemented with 2% (*v*/*v*) of FBS, and Edicotinib in a medium with 10% (*v*/*v*) of FBS. Edicotinib was used to pharmacologically inhibit macrophage activity as previously reported [23]. DMSO concentrations ranging from 0.1% to 0.5% (*v*/*v*) were evaluated, and no cytotoxic effects were observed at these concentrations. All experimental groups, including the main controls, were exposed to the same final concentration (0.5%) to exclude any potential vehicle-related effects.

### 4.4. Non-Linear Regression for IC_50_ Determination

The half-maximal inhibitory concentrations (IC_50_) for Dabrafenib and Trametinib were calculated using Cell Counting Kit-8 (CCK8; Dojindo, Kumamoto, Japan) and defined by a non-linear regression (curve fit) analysis.

### 4.5. Macrophage–ATC Co-Cultures

Macrophages and ATC cells were plated separately in two independent compartments of a 2D transwell system as described by Pinto et al. [22]. The 2D transwell system enables indirect communication between ATC cells and THP-1-derived macrophages, which are plated in different compartments. A serum gradient was maintained constant across all experimental groups within each assay ensuring that serum concentration was not a variable influencing the observed treatment effects, and to reproduce the conditions established in our previous study [22]. THP-1-derived macrophages were cultured in a medium containing 10% FBS, whereas ATC cells were cultured in 2% FBS during DT treatment to minimise the influence of serum-derived growth factors in DT efficacy. The cell seeding densities were optimised according to the culture surface area and the intrinsic biological characteristics of each cell line to ensure comparable cell confluence and therapeutic responses across experimental conditions.

### 4.6. Cell Viability Assays

Cell viability assessment throughout time, upon drug exposure, was performed using Trypan Blue exclusion method. ATC cells were plated in 12-well plates at a density of 3.5 × 10^4^ per well. After 24 h, ATC cells were exposed to DT (Dabrafenib—10 nM, Trametinib—5 nM) and to THP-1-derived macrophages (9 × 10^4^ cells/insert; 0.4 µm pore, Corning), with or without 10 µM of Edicotinib [23]. THP-1-derived macrophages were exposed to Edicotinib simultaneously with the exposure of ATC cells to DT and co-culture. The drugs were maintained in the culture medium throughout the duration of the assay. Then, viable ATC cells were counted at 24, 48 and 72 h after drug exposure.

### 4.7. Invasion Assays

To evaluate the effects of DT and macrophages on ATC cells invasiveness, invasion assays were performed, with cells exposed to DT (Dabrafenib—10 nM, Trametinib—5 nM) and macrophages for 24 h. The invasion methodology was performed as previously described by Pinto et al. [22], with the addition of DT and Edicotinib (10 µM), in their respective assays. Specifically, ATC cells (T235—9 × 10^4^; T238—7.5 × 10^4^) were seeded into Matrigel-coated inserts (8 μm pore size, Corning) in DT-supplemented culture medium and co-cultured with THP-1-derived macrophages (2 × 10^5^/well) with or without Edicotinib, as previously mentioned. Image acquisition and data analysis were performed using an Imager Z1 epifluorescence microscope (Zeiss, Oberkochen, Germany) connected to Cytovision software (version 7.4; Leica Biosystems, Nussloch, Germany). Images were processed in Fiji/ImageJ (https://imagej.net/ij/ accessed on 20 August 2026, version 1.54p).

### 4.8. Immunofluorescence for Actin and Vimentin

To evaluate the actin cytoskeleton organisation and vimentin expression in ATC cells upon co-culture with macrophages and exposure to DT (Dabrafenib—10 nM, Trametinib—5 nM), actin filaments were stained using a phalloidin staining protocol (Sigma, St. Louis, MO, USA) and vimentin was detected using mouse anti-vimentin primary antibody (#550513, BD pharmingen, Franklin Lakes, NJ, USA). The detailed methodology is provided in the Supplementary Methods.

### 4.9. qRT-PCR

To analyse *SPRY4* expression, cells were plated in a confluence of 8.7 × 10^4^/per well in 6-well plates. After 72 h of DT (Dabrafenib—10 nM, Trametinib—5 nM) and macrophage exposure, RNA was extracted from ATC cells using RNeasy (Qiagen, Hilden, Germany) according to the manufacturer’s protocol, and quantified for cDNA synthesis. RT-qPCR was done according to SYBR Green protocol, using a QuantStudio 5 Real-time PCR System (Applied Biosystems, Waltham, CA, USA). The relative expression levels of *SPRY4* gene were determined using the ΔΔCt method, with *HPRT1* as the endogenous control gene. Fold change was also calculated [FC = 2^ΔΔCt^]. Primers are described by Pinto et al. [22].

### 4.10. Western Blot

To investigate the effects of DT (Dabrafenib—10 nM, Trametinib—5 nM) and macrophages in SPRY4 protein expression and MAPK pathway activation, Western blot analyses for SPRY4, ERK1/2, and phosphorylated ERK1/2 (pERK1/2) were performed. After 72 h of DT and macrophage exposure, protein was extracted from ATC cells and quantified. The following primary antibodies were used: anti-SPRY4 rabbit monoclonal antibody (clone EPR12127, #ab176337, Abcam, Cambridge, UK); anti-ERK1/2 (144-64587-50; RayBiotech, Norcross, GA, USA), and anti-pERK1/2 (612358; BD Biosciences, Franklin Lakes, NJ, USA). To detect the control proteins, primary antibodies for β-actin (clone AC-15, A5441, Sigma-Aldrich, St. Louis, MO, USA) and α-tubulin (clone B-5-1-2; #T5168, Sigma-Aldrich, St. Louis, MO, USA) were used. Band area quantification was performed using Fiji image J (https://imagej.net/ij/ accessed on 20 August 2026, version 1.54p). Conditions and antibodies are presented in Table S6.

### 4.11. Immunohistochemistry for PD-L1 in ATC Cell Lines

Immunohistochemistry (IHC) was performed to evaluate the effects of ATC–macrophage interactions and DT in PD-L1 (programmed death-ligand 1) expression in ATC cell lines. Briefly, after 72h of co-culture between cells with/without DT, ATC cells were isolated using PBS + 4 mM EDTA and detached gently using a cell scraper. The cellular pellets were transformed into formalin-fixed paraffin-embedded (FFPE) cell blocks. IHC was performed using Ventana Benchmark Ultra (Roche Diagnostics, Lisbon, Portugal) for PD-L1 (clone 22C3, M3653, Dako, Glostrup, Denmark). Data analysis was performed by an expert pathologist. IHC staining was qualitatively interpreted based on the expression (positive and negative) and the staining intensity (low, moderate and high). Images were analysed using Aperio ImageScope (version 12.4; Leica Biosystems, Nussloch, Germany). Conditions and antibodies are detailed in Table S6.

### 4.12. Flow Cytometry

To further clarify the effects of ATC–macrophage interactions, DT (Dabrafenib—10 nM, Trametinib—5 nM) and Edicotinib (10 µM) in macrophage polarisation, we performed flow cytometry for CD80 and CD163. Cells were plated and isolated as described above. For CD80 and CD163 staining, anti-CD80, conjugated with allophycocyanin, (APC) and anti-CD163, conjugated with APC (Biolegend, San Diego, CA, USA), were used. A minimum of 10,000 total events were acquired for each sample. Conditions and antibodies are presented in Table S6.

### 4.13. Immunohistochemistry Analysis of ATC from Patients Under DT

Biopsy or surgical samples from eleven ATC patients under DT were used. All ATC diagnoses were confirmed by pathologists specialised in endocrine tumour pathology. IHC was performed for CD68 (clone KP1, 790-2931, Ventana, Oro Valley, AZ, USA) and CD163 (clone MRQ-26, 760-4437, CELL MARQUE, Rocklin, CA, USA), using Ventana Benchmark Ultra (Roche Diagnostics, Lisbon, Portugal). IHC staining was qualitatively interpreted based on the expression (positive and negative) and the staining intensity (low, moderate and high). Images were analysed using Aperio ImageScope (version 12.4; Leica Biosystems). Conditions and antibodies are detailed in Table S6.

### 4.14. Statistical Analyses

The *in vitro* quantitative experimental data were analysed using GraphPad Prism version 8.0.1. (Boston, MA, USA). Data are presented as mean (standard deviation) from at least three independent biological assays and at least two technical replicates per condition. Statistical analyses were done according to the type of assay performed. Cell viability assays were analysed using one-way or two-way ANOVA (followed by Tukey’s multiple comparison test), as appropriate. Invasion assays, gene expression analyses, and protein expression quantification were analysed using one-way ANOVA (Tukey’s test). Flow cytometry analyses were performed using one-way ANOVA (Tukey’s test) or unpaired parametric *t* test, as appropriate. Non-linear regression analysis was used for the IC_50_ determination. Data normality was assessed using the Shapiro–Wilk test prior to parametric analyses. A *p*-value < 0.05 was considered statistically significant.

## Data Availability

The original contributions presented in this study are included in the article/Supplementary Materials. Further inquiries can be directed to the corresponding author.

## Supplementary Materials

The following supporting information can be downloaded at: https://www.mdpi.com/article/10.3390/ijms27177691/s1. Reference [43] are cited in the Supplementary Materials.

## References

[1] Simões-Pereira J., Capitão R., Limbert E., Leite V. (2019). Anaplastic Thyroid Cancer: Clinical Picture of the Last Two Decades at a Single Oncology Referral Centre and Novel Therapeutic Options. Cancers.

[2] Bible K.C., Kebebew E., Brierley J., Brito J.P., Cabanillas M.E., Clark T.J., Di Cristofano A., Foote R., Giordano T., Kasperbauer J. (2021). 2021 American Thyroid Association Guidelines for Management of Patients with Anaplastic Thyroid Cancer. Thyroid.

[3] Baloch Z.W., Asa S.L., Barletta J.A., Ghossein R.A., Juhlin C.C., Jung C.K., LiVolsi V.A., Papotti M.G., Sobrinho-Simões M., Tallini G. (2022). Overview of the 2022 WHO Classification of Thyroid Neoplasms. Endocr. Pathol..

[4] Wirth L.J., Brose M.S., Sherman E.J., Licitra L., Schlumberger M., Sherman S.I., Bible K.C., Robinson B., Rodien P., Godbert Y. (2021). Open-Label, Single-Arm, Multicenter, Phase II Trial of Lenvatinib for the Treatment of Patients with Anaplastic Thyroid Cancer. J. Clin. Oncol..

[5] Prasongsook N., Kumar A., Chintakuntlawar A.V., Foote R.L., Kasperbauer J., Molina J., Garces Y., Ma D., Neben Wittich M.A., Rubin J. (2017). Survival in Response to Multimodal Therapy in Anaplastic Thyroid Cancer. J. Clin. Endocrinol. Metab..

[6] Subbiah V., Cabanillas M.E., Kreitman R.J., Wainberg Z.A., Cho J.Y., Keam B., Schellens J.H.M., Soria J.C., Wen P.Y., Zielinski C. (2018). Dabrafenib and Trametinib Treatment in Patients with Locally Advanced or Metastatic BRAF V600–Mutant Anaplastic Thyroid Cancer. J. Clin. Oncol..

[7] da Silva T.N., Rodrigues R., Saramago A., Pires C., Rito M., Horta M., Martins C., Leite V., Cavaco B.M. (2023). Target Therapy for BRAF Mutated Anaplastic Thyroid Cancer: A Clinical and Molecular Study. Eur. J. Endocrinol..

[8] Subbiah V., Kreitman R.J., Wainberg Z.A., Cho J.Y., Schellens J.H.M., Soria J.C., Wen P.Y., Zielinski C.C., Cabanillas M.E., Boran A. (2022). Dabrafenib plus Trametinib in Patients with BRAF V600E-Mutant Anaplastic Thyroid Cancer: Updated Analysis from the Phase II ROAR Basket Study. Ann. Oncol..

[9] Lorimer C., Cheng L., Chandler R., Garcez K., Gill V., Graham K., Grant W., Sardo Infirri S., Wadsley J., Wall L. (2023). Dabrafenib and Trametinib Therapy for Advanced Anaplastic Thyroid Cancer—Real-World Outcomes from UK Centres. Clin. Oncol..

[10] Bueno F., Smulever A., Califano I., Guerra J., Del Grecco A., Carrera J.M., Giglio R., Rizzo M., Lingua A., Voogd A. (2023). Dabrafenib plus Trametinib Treatment in Patients with Anaplastic Thyroid Carcinoma: An Argentinian Experience. Endocrine.

[11] Cabanillas M.E., Dadu R., Iyer P., Wanland K.B., Busaidy N.L., Ying A., Gule-Monroe M., Wang J.R., Zafereo M., Hofmann M.C. (2020). Acquired Secondary RAS Mutation in BRAFV600E-Mutated Thyroid Cancer Patients Treated with BRAF Inhibitors. Thyroid.

[12] Hofmann M.C., Kunnimalaiyaan M., Wang J.R., Busaidy N.L., Sherman S.I., Lai S.Y., Zafereo M., Cabanillas M.E. (2022). Molecular Mechanisms of Resistance to Kinase Inhibitors and Redifferentiation in Thyroid Cancers. Endocr. Relat. Cancer.

[13] Wu P.K., Park J.I. (2015). MEK1/2 Inhibitors: Molecular Activity and Resistance Mechanisms. Semin. Oncol..

[14] Nathanson K.L., Martin A.M., Wubbenhorst B., Greshock J., Letrero R., D’Andrea K., O’Day S., Infante J.R., Falchook G.S., Arkenau H.T. (2013). Tumor Genetic Analyses of Patients with Metastatic Melanoma Treated with the BRAF Inhibitor Dabrafenib (GSK2118436). Clin. Cancer Res..

[15] Irvine M., Stewart A., Pedersen B., Boyd S., Kefford R., Rizos H. (2018). Oncogenic PI3K/AKT Promotes the Step-Wise Evolution of Combination BRAF/MEK Inhibitor Resistance in Melanoma. Oncogenesis.

[16] Ryder M., Ghossein R.A., Ricarte-Filho J.C.M., Knauf J.A., Fagin J.A. (2008). Increased Density of Tumor-Associated Macrophages Is Associated with Decreased Survival in Advanced Thyroid Cancer. Endocr. Relat. Cancer.

[17] Stempin C.C., Geysels R.C., Park S., Palacios L.M., Volpini X., Motran C.C., Rodríguez E.V.A., Nicola J.P., Cheng S.Y., Pellizas C.G. (2021). Secreted Factors by Anaplastic Thyroid Cancer Cells Induce Tumor-Promoting M2-like Macrophage Polarization through a Tim3-Dependent Mechanism. Cancers.

[18] Giannini R., Moretti S., Ugolini C., MacErola E., Menicali E., Nucci N., Morelli S., Colella R., Mandarano M., Sidoni A. (2019). Immune Profiling of Thyroid Carcinomas Suggests the Existence of Two Major Phenotypes: An ATC-Like and a PDTC-Like. J. Clin. Endocrinol. Metab..

[19] Cho J.W., Kim W.W., Lee Y., Jeon M.J., Kim W.G., Song D.E., Park Y., Chung K.W., Hong S.J., Sung T.Y. (2019). Impact of Tumor-Associated Macrophages and BRAFV600E Mutation on Clinical Outcomes in Patients with Various Thyroid Cancers. Head Neck.

[20] Lee M., Untch B.R., Xu B., Ghossein R., Han C., Kuo F., Valero C., Nadeem Z., Patel N., Makarov V. (2021). Genomic and Transcriptomic Correlates of Thyroid Carcinoma Evolution after BRAF Inhibitor Therapy. Mol. Cancer Res..

[21] Pan H., Xu R., Zhang Y. (2024). Role of SPRY4 in Health and Disease. Front. Oncol..

[22] Pinto A.T., Pojo M., Rodrigues R., Sousa D.P., Matthiesen R., Carvalho A.S., Beck H.C., Pires C., Eduardo R., Pereira J.S. (2023). SPRY4 as a Potential Mediator of the Anti-Tumoral Role of Macrophages in Anaplastic Thyroid Cancer Cells. Cancers.

[23] Gharzeddine K., Prieto C.G., Malier M., Hennot C., Grespan R., Yamaryo-Botté Y., Botté C.Y., Thomas F., Laverriere M.H., Girard E. (2024). Metabolic Reprogramming of Hypoxic Tumor-Associated Macrophages through CSF-1R Targeting Favors Treatment Efficiency in Colorectal Cancers. J. Immunother. Cancer.

[24] Caillou B., Talbot M., Weyemi U., Pioche-Durieu C., Ghuzlan A., Bidart J.M., Chouaib S., Schlumberger M., Dupuy C. (2011). Tumor-Associated Macrophages (TAMs) Form an Interconnected Cellular Supportive Network in Anaplastic Thyroid Carcinoma. PLoS ONE.

[25] Chen Y., Zhong J., Sun W., Yan W., Wang C., Liu W., Lin X., Zou Z. (2022). BRAF Inhibitor Resistance in Melanoma: Mechanisms and Alternative Therapeutic Strategies. Curr. Treat. Options Oncol..

[26] Wang S., Wang J., Chen Z., Luo J., Guo W., Sun L., Lin L. (2024). Targeting M2-like Tumor-Associated Macrophages Is a Potential Therapeutic Approach to Overcome Antitumor Drug Resistance. npj Precis. Oncol..

[27] Olson O.C., Kim H., Quail D.F., Foley E.A., Joyce J.A. (2017). Tumor-Associated Macrophages Suppress the Cytotoxic Activity of Antimitotic Agents. Cell Rep..

[28] Ruffell B., Coussens L.M. (2015). Macrophages and Therapeutic Resistance in Cancer. Cancer Cell.

[29] Tennis M.A., Van Scoyk M.M., Freeman S.V., Vandervest K.M., Nemenoff R.A., Winn R.A. (2010). Sprouty-4 Inhibits Transformed Cell Growth, Migration and Invasion, and Epithelial-Mesenchymal Transition, and Is Regulated by Wnt7a through PPARγ in Non-Small Cell Lung Cancer. Mol. Cancer Res..

[30] Masoumi-Moghaddam S., Amini A., Morris D.L. (2014). The Developing Story of Sprouty and Cancer. Cancer Metast. Rev..

[31] Cabrita M.A., Christofori G. (2008). Sprouty Proteins, Masterminds of Receptor Tyrosine Kinase Signaling. Angiogenesis.

[32] Stutvoet T.S., Kol A., de Vries E.G.E., de Bruyn M., Fehrmann R.S.N., Terwisscha van Scheltinga A.G.T., de Jong S. (2019). MAPK Pathway Activity Plays a Key Role in PD-L1 Expression of Lung Adenocarcinoma Cells. J. Pathol..

[33] Jiang X., Zhou J., Giobbie-Hurder A., Wargo J., Hodi F.S. (2013). The Activation of MAPK in Melanoma Cells Resistant to BRAF Inhibition Promotes PD-L1 Expression That Is Reversible by MEK and PI3K Inhibition. Clin. Cancer Res..

[34] Angell T.E., Lechner M.G., Jang J.K., Correa A.J., LoPresti J.S., Epstein A.L. (2014). BRAFV600E in Papillary Thyroid Carcinoma Is Associated with Increased Programmed Death Ligand 1 Expression and Suppressive Immune Cell Infiltration. Thyroid.

[35] Chintakuntlawar A.V., Rumilla K.M., Smith C.Y., Jenkins S.M., Foote R.L., Kasperbauer J.L., Morris J.C., Ryder M., Alsidawi S., Hilger C. (2017). Expression of PD-1 and PD-L1 in Anaplastic Thyroid Cancer Patients Treated with Multimodal Therapy: Results from a Retrospective Study. J. Clin. Endocrinol. Metab..

[36] Menicali E., Guzzetti M., Morelli S., Moretti S., Puxeddu E. (2021). Immune Landscape of Thyroid Cancers: New Insights. Front. Endocrinol..

[37] Na Y.R., Hong J.H., Lee M.Y., Jung J.H., Jung D., Kim Y.W., Son D., Choi M., Kim K.P., Seok S.H. (2015). Proteomic Analysis Reveals Distinct Metabolic Differences between Granulocyte-Macrophage Colony Stimulating Factor (GM-CSF) and Macrophage Colony Stimulating Factor (M-CSF) Grown Macrophages Derived from Murine Bone Marrow Cells. Mol. Cell. Proteom..

[38] Shang Q., Zhang P., Lei X., Du L., Qu B. (2025). Insights into CSF-1/CSF-1R Signaling: The Role of Macrophage in Radiotherapy. Front. Immunol..

[39] Hamilton T.A., Zhao C., Pavicic P.G., Datta S. (2014). Myeloid Colony-Stimulating Factors as Regulators of Macrophage Polarization. Front. Immunol..

[40] Wang Q., Wang J., Xu K., Luo Z. (2024). Targeting the CSF1/CSF1R Signaling Pathway: An Innovative Strategy for Ultrasound Combined with Macrophage Exhaustion in Pancreatic Cancer Therapy. Front. Immunol..

[41] Zhao Y., Zhao B., Wang X., Guan G., Xin Y., Sun Y.D., Wang J.H., Guo Y., Zang Y.J. (2019). Macrophage Transcriptome Modification Induced by Hypoxia and Lactate. Exp. Ther. Med..

[42] Smirnova T., Spertini C., Spertini O. (2021). Csf1r Inhibition Combined with Gm-csf Reprograms Macrophages and Disrupts Protumoral Interplays with Aml Cells. Cancers.

[43] Landa I., Pozdeyev N., Korch C., Marlow L.A., Smallridge R.C., Copland J.A., Henderson Y.C., Lai S.Y., Clayman G.L., Onoda N. (2019). Comprehensive Genetic Characterization of Human Thyroid Cancer Cell Lines: A Validated Panel for Preclinical Studies. Clin. Cancer Res..

