# Supplementary material for: Macrophages as Drivers of Resistance to Dabrafenib Plus Trametinib Therapy in Anaplastic Thyroid Cancer
Source: Int J Mol Sci. 2026 Aug 27;27(17):7691. doi: 10.3390/ijms27177691 (PMC13565890; doi:10.3390/ijms27177691)
Supplement: Supplementary file 1 [file ijms-27-07691-s001.zip › ijms-4444863-supplementary.pdf]

Macrophages as drivers of resistance to Dabrafenib plus Trametinib therapy in Anaplastic Thyroid Cancer

Ricardo Rodrigues[1,2], Beatriz Garcia[1,3], Miguel Rito[4,5], Rúben Roque[4,6], Teresa Pereira[4], Sónia Morgado[4], Vanessa Tavares[4], Tiago Nunes da Silva[1,7], Valeriano Leite[1,7], Branca Maria Cavaco[1,*]

[1] Unidade de Investigação em Patobiologia Molecular (UIPM), Instituto Português de Oncologia de Lisboa Francisco Gentil (IPOLFG), Rua Professor Lima Basto, 1099-023 Lisboa, Portugal; rmrodrigues@ipolisboa.min-saude.pt (R.R.); ba.garcia@campus.fct.unl.pt (B.G.); tlnsilva@ipolisboa.min-saude.pt (T.N.S.); vleite@ipolisboa.min-saude.pt (V.L.)

[2] Faculdade de Ciências Médicas, Universidade NOVA de Lisboa, Campo dos Mártires da Pátria, 130, 1169-056 Lisboa, Portugal

[3] Faculdade de Ciências e Tecnologia, Universidade NOVA de Lisboa, Campus da Caparica, 2829-516 Caparica, Portugal

[4] Serviço de Anatomia Patológica, Instituto Português de Oncologia de Lisboa Francisco Gentil (IPOLFG), Rua Professor Lima Basto, 1099-023 Lisboa, Portugal; mrito@ipolisboa.min-saude.pt (M.R.); rroque@ipolisboa.min-saude.pt (R.R.); tpereira@ipolisboa.min-saude.pt (T.P.); smorgado@ipolisboa.min-saude.pt (S.M.); vtavares@ipolisboa.min-saude.pt (V.T.)

[5] Instituto Anatomia Patológica, Faculdade de Medicina da Universidade de Lisboa, Av. Prof. Egas Moniz, 1649-028 Lisboa, Portugal

[6] Escola Superior de Saúde de Lisboa, Politécnico de Lisboa, Estrada de Benfica, 529, 1549-020 Lisboa, Portugal

[7] Serviço de Endocrinologia, Instituto Português de Oncologia de Lisboa Francisco Gentil (IPOLFG), Rua Professor Lima Basto, 1099-023 Lisboa, Portugal

* Correspondence: bcavaco@ipolisboa.min-saude.pt

Supplementary data

Supplementary Methods

Immunofluorescence for actin and vimentin

Cells were seeded onto glass slides coated with 0.2% (w/v) gelatine from porcine skin (G-1890, Sigma-Aldrich, St. Louis, MO, USA), placed in 12-well plates. After 72 hours, the culture medium was removed, and cells were washed with PBS and fixed with 4% (w/v) paraformaldehyde (PFA; Sigma-Aldrich, St. Louis, Missouri, USA) for 20 minutes. Actin staining was performed using phalloidin-FITC (Sigma-Aldrich, St. Louis, Missouri, USA), diluted 1:500 and incubated for 1 hour and 30 minutes at room temperature in the dark under agitation. For vimentin staining, cells were incubated overnight at 4°C with a 1:100 dilution of mouse anti-vimentin primary antibody (#550513, BD pharmingen, Franklin Lakes, New Jersey, USA). The secondary antibody, goat anti-mouse Alexa Fluor 488 (A-10680, Invitrogen, Carlsbad, California, USA), was then applied. Finally, slides were mounted with DAPI-Vectashield (Vector Laboratories, Newark, California, USA). Image acquisition and data analysis were performed using an Imager Z1 epifluorescence microscope (Zeiss, Oberkochen, Germany) connected to Cytovision software (version 7.4; Leica Biosystems, Nussloch, Germany). Images were processed in Fiji/ImageJ (https://imagej.net/ij/, version 1.54p).

Supplementary Figures

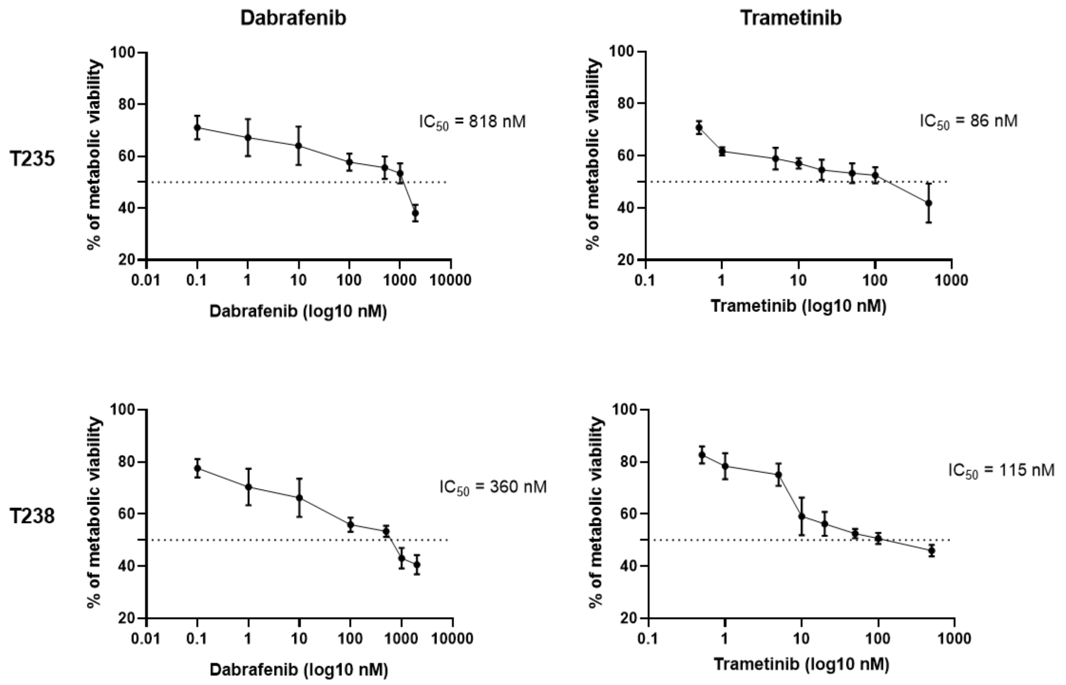

Figure S1. Dose response inhibition curves for Dabrafenib and Trametinib for T235 and T238 cells. The dose response inhibition curves were performed from three independent biological assays, each with three technical replicates, using [Inhibitor] vs. response - Variable slope (four parameters) in Graphpad prism version 8.0.1. The values are represented as mean (standard deviation). nM, nanomolar.

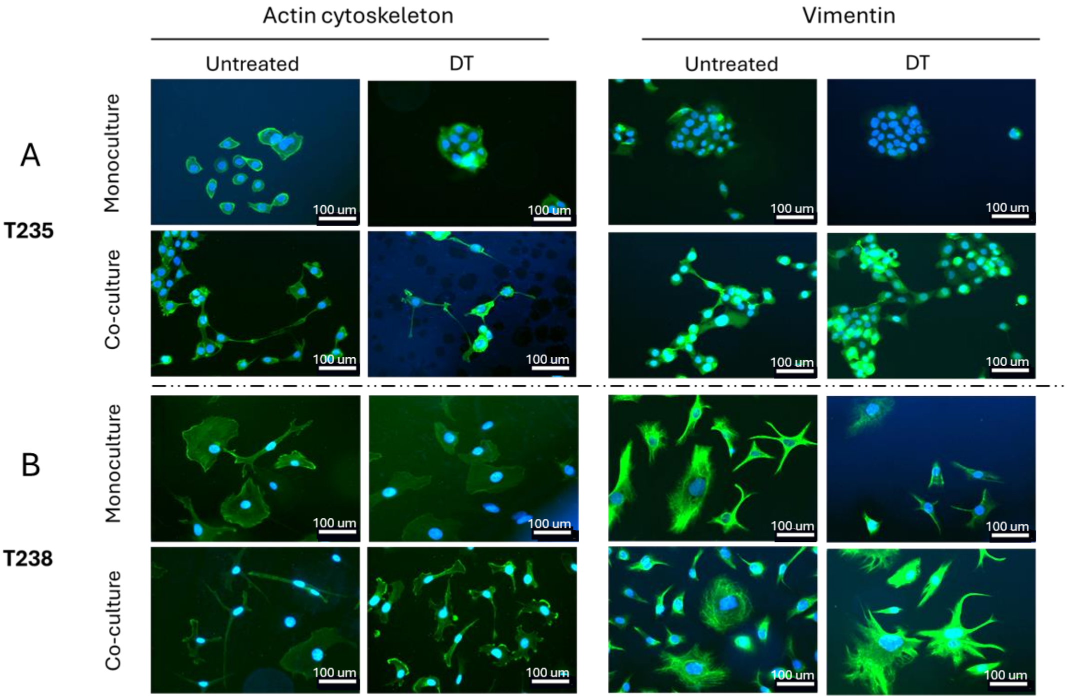

Figure S2. Actin cytoskeleton organization and vimentin expression of ATC cell lines in mono and co-cultures, with or without Dabrafenib plus Trametinib. Representative images of the actin cytoskeleton and vimentin expression for (A) T235 and (B) T238, from three independent biological assays, each with two technical replicates. Cells were fixed and stained for F-actin (green) or vimentin (green), and the nucleus (blue). Image scales at 100 μm.

a) T235

i) SPRY4

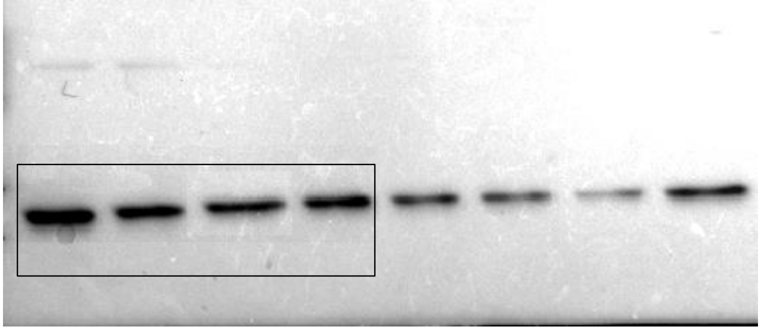

β-actin

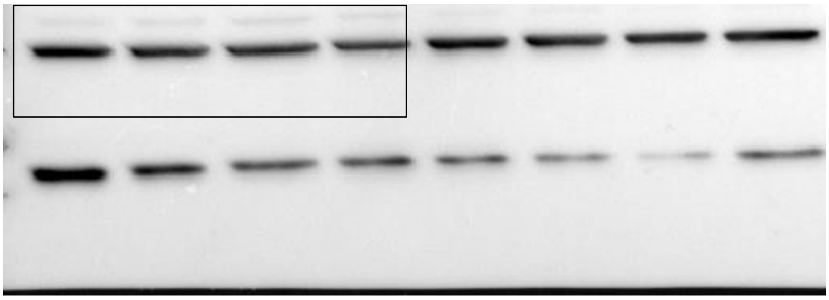

ii) ERK1/2

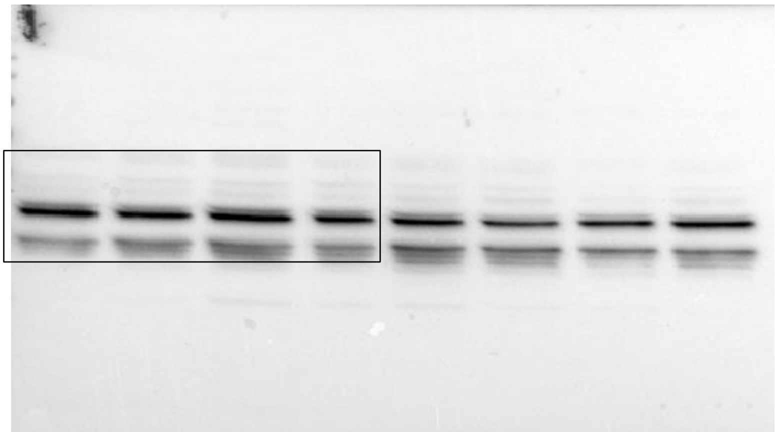

α-tubulin

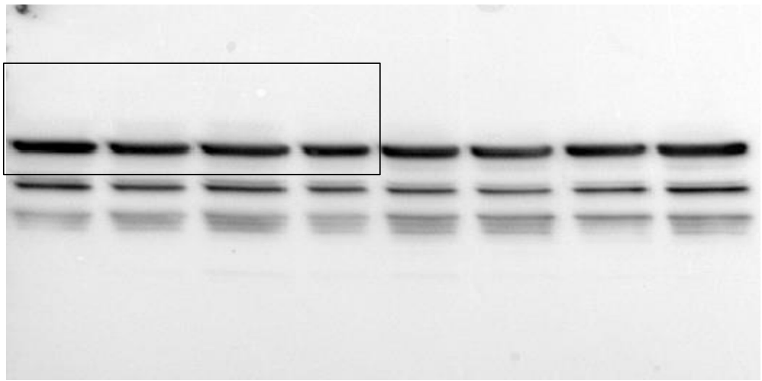

iii) pERK1/2

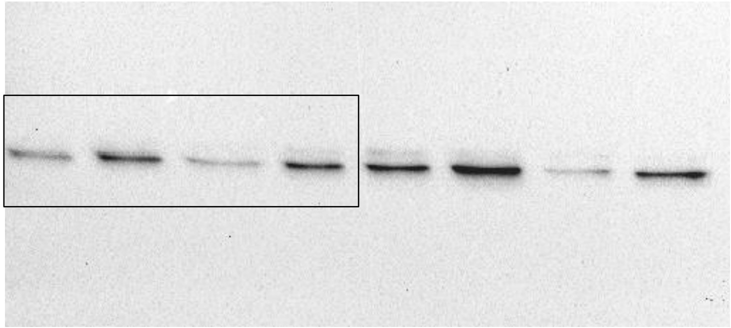

α-tubulin

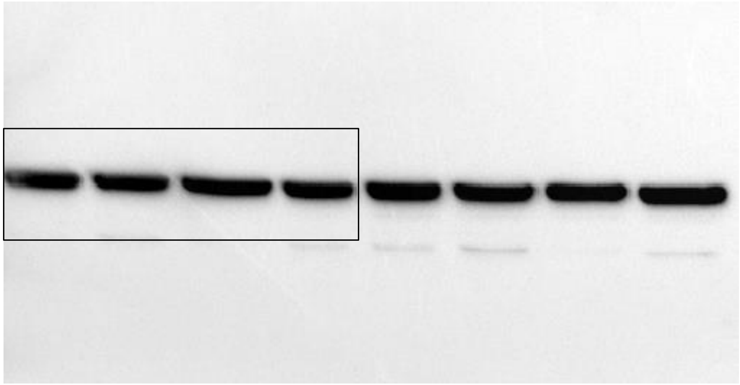

b) T238

i) SPRY4

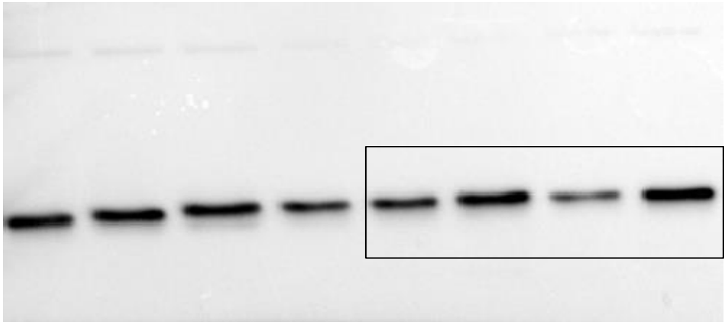

β-actin

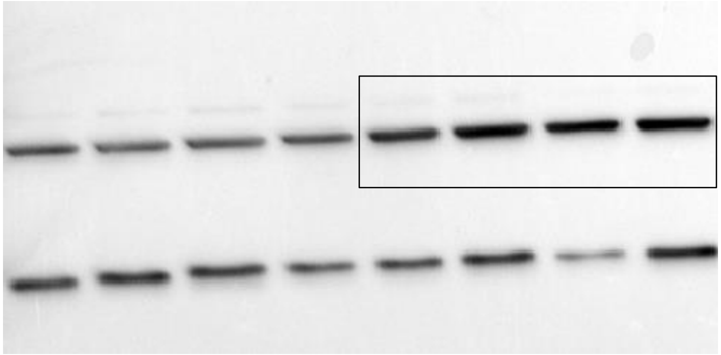

ii) ERK1/2

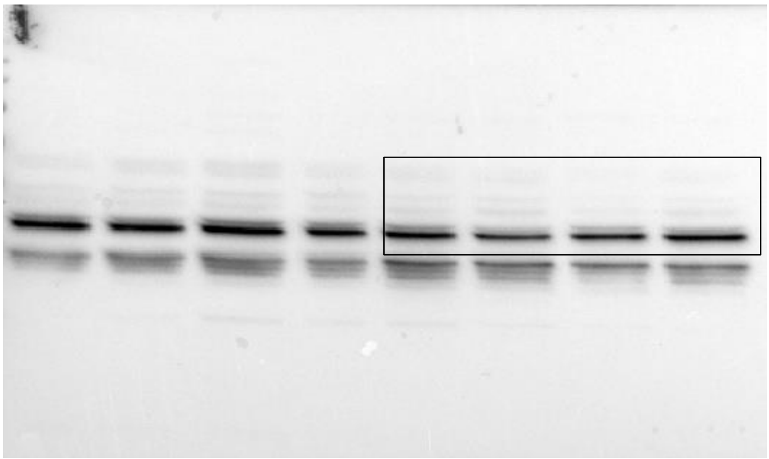

α-tubulin

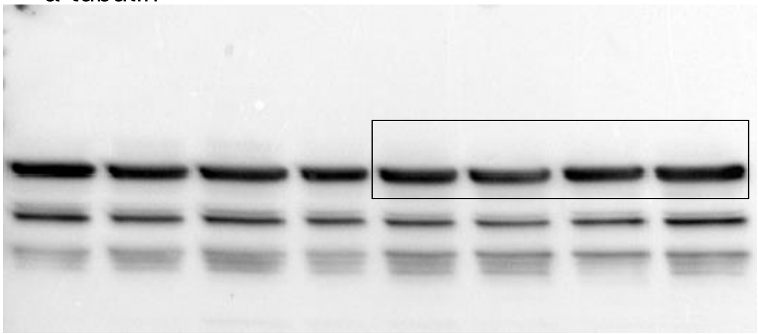

iii) pERK1/2

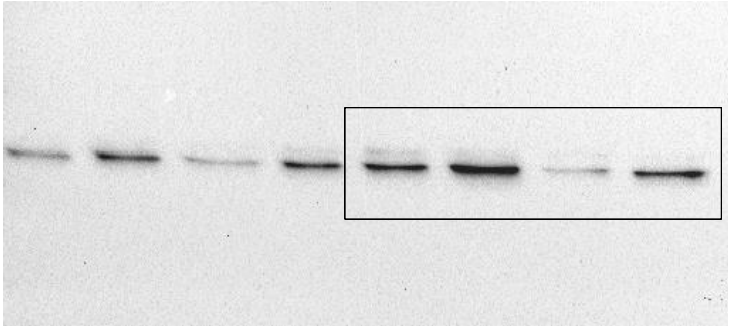

α-tubulin

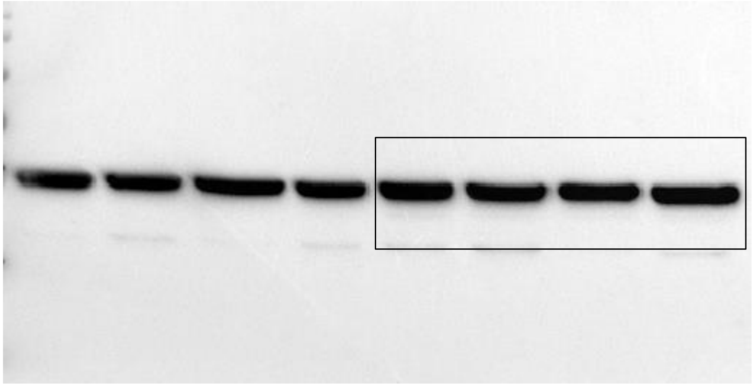

Figure S3. Full Western blot membranes for SPRY4, ERK1/2 and pERK1/2, and respective control proteins (β-actin and α-tubulin) for (A) T235 and (B) T238 cells.

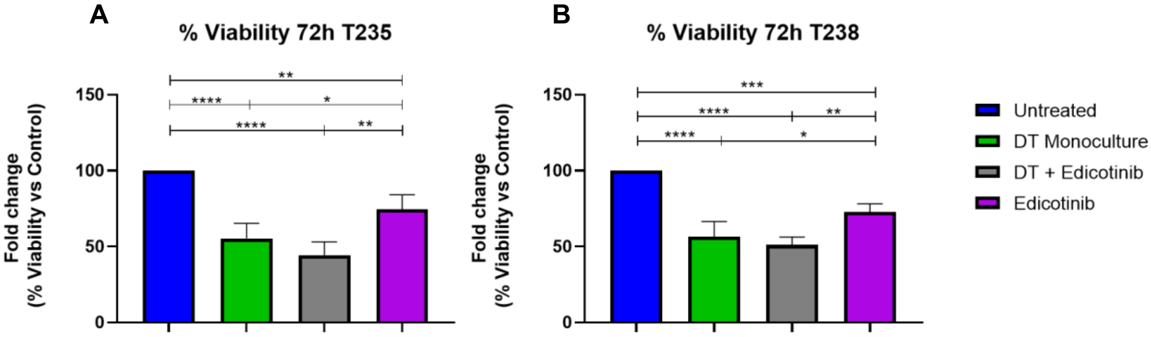

Figure S4. Cell viability of T235 and T238 in monocultures under DT and Edicotinib in monotherapy and combined therapy. Viability assays of (A) T235 and (B) T238 for the % of viability at 72h. The normalisation control (untreated) was defined as equal to 100. The values are represented as mean (standard deviation) from four independent biological assays, each with two technical replicates, and analysed using one-way ANOVA. * $p<0.05$, ** $p<0.01$, *** $p<0.001$, **** $p<0.0001$. Plots: blue – untreated monoculture, green – DT monoculture, grey – DT + Edicotinib monoculture, purple – Edicotinib monoculture.

Supplementary Tables

Table S1. Mutational characteristics of the ATC cell lines and IC_{50} for Dabrafenib and Trametinib determined using the CCK8 assay

**ATC cell lines**	**Mutated genes**					**Dabrafenib IC_{50} (nM)**	**Trametinib IC_{50} (nM)**
	BRAF	***PIK3CA***	***TP53***	***TERTp***	***CDKN2A***		
T235	p.V600E	wt	p.L194R	C228T	wt	818	86
T238	p.V600E	p.E542K	p.S183ter	C228T	p.L63R	360	115

IC_{50} values are represented as mean from three independent samples. ATC, Anaplastic thyroid cancer; wt, wild-type genotype; $IC_{50,}$ half maximal inhibitory concentration; nM, nanomolar, CCK8, cell-counting kit 8. Mutational analysis retrieved from Landa *et al.* [43].

Table S2. Data overview from Viability assays in the ATC cells under DT therapy in mono and co-cultures for 24, 48 and 72h of exposure

Cell line	Timepoint	Mean total number of viable cells (n = 4)				Two-Way ANOVA statistics						Mean % viability *vs* U mono (n = 4)		
		U mono	U co	DT mono	DT co	U mono vs U co	U mono vs DT mono	U mono vs DT co	U co vs DT mono	U co vs DT co	DT mono vs DT co	U co	DT mono	DT co
T235	24h	73958	75833	50625	78750	ns	**	ns	***	ns	**	102.6	68.5	106.4
	48h	143542	143750	78958	154375	ns	**	ns	**	ns	***	100.3	55.2	107.9
	72h	219375	224167	144375	257500	ns	*	*	*	ns	**	102.2	65.6	117.3
T238	24h	72917	63750	48958	96250	ns	**	****	**	**	***	87.3	67.07	132.01
	48h	134167	95208	74375	158542	ns	**	ns	ns	**	**	71.5	55.8	118.6
	72h	206042	162500	102500	246250	*	**	*	ns	*	**	78.7	49.8	119.7

The data is represented as the mean value of four independent assays analysed with two-way ANOVA for total number of viable cells. * $p < 0.05$; ** $p < 0.01$; *** $p < 0.001$; **** $p < 0.0001$. U - Untreated, DT - Dabrafenib plus Trametinib, Mono - monoculture, Co - co-culture

Table S3. Overview of the immunohistochemistry data analysis in the ATC cell blocks

**Cell line**	**Condition**	**PD-L1 expression**
T235	U mono	0
T235	U co	+
T235	DT mono	-
T235	DT co	+ +
T238	U mono	0
T238	U co	+ +
T238	DT mono	-
T238	DT co	+

For PD-L1 expression, data is represented in symbols (0, control; +, low positive expression; ++, intermediate positive expression; -, expression lower than 0) and it was compared to the control/untreated monoculture (0). Markers were analysed by the same expert pathologist. Mono – monoculture; U mono - untreated monoculture; U co – untreated co-culture; DT mono – DT monoculture; DT co - DT co-culture.

Table S4. Data overview from Viability assays in the ATC cells under DT in mono and co-cultures, with or without Edicotinib.

Cell line	Timepoint	Mean total number of viable cells (n=3)				Mean % Viability *vs* DT mono (n = 3)			
				Two-way ANOVA				ANOVA	
		Conditions	Counts	Conditions	p-value	Conditions	Mean %	Conditions	p-value
T235	24h	DT mono	70417	DT mono vs DT co	**	DT co	168	DT mono vs DT co	****
		DT co	117917	DT mono vs DT co + Edicotinib	ns	DT co + Edicotinib	105	DT mono vs DT co + Edicotinib	ns
		DT co + Edicotinib	73750	DT co vs DT co + Edicotonib	*			DT co vs DT co + Edicotonib	****
	48h	DT mono	149583	DT mono vs DT co	ns	DT co	120	DT mono vs DT co	**
		DT co	179583	DT mono vs DT co + Edicotinib	ns	DT co + Edicotinib	99	DT mono vs DT co + Edicotinib	ns
		DT co + Edicotinib	147500	DT co vs DT co + Edicotonib	ns			DT co vs DT co + Edicotonib	**
	72h	DT mono	163333	DT mono vs DT co	**	DT co	151	DT mono vs DT co	***
		DT co	245000	DT mono vs DT co + Edicotinib	ns	DT co + Edicotinib	102	DT mono vs DT co + Edicotinib	ns
		DT co + Edicotinib	165833	DT co vs DT co + Edicotonib	**			DT co vs DT co + Edicotonib	**
T238	24h	DT mono	58333	DT mono vs DT co	*	DT co	208	DT mono vs DT co	ns
		DT co	117500	DT mono vs DT co + Edicotinib	ns	DT co + Edicotinib	171	DT mono vs DT co + Edicotinib	ns
		DT co + Edicotinib	96667	DT co vs DT co + Edicotonib	ns			DT co vs DT co + Edicotonib	ns
	48h	DT mono	105000	DT mono vs DT co	ns	DT co	142	DT mono vs DT co	*
		DT co	146667	DT mono vs DT co + Edicotinib	ns	DT co + Edicotinib	98	DT mono vs DT co + Edicotinib	ns
		DT co + Edicotinib	103750	DT co vs DT co + Edicotonib	ns			DT co vs DT co + Edicotonib	*
	72h	DT mono	136250	DT mono vs DT co	*	DT co	144	DT mono vs DT co	***
		DT co	196667	DT mono vs DT co + Edicotinib	ns	DT co + Edicotinib	103	DT mono vs DT co + Edicotinib	ns
		DT co + Edicotinib	140833	DT co vs DT co + Edicotonib	*			DT co vs DT co + Edicotonib	***

Total cell counts were analysed by two-way ANOVA (Tukey's multiple-comparisons test). Viability was analysed by one-way ANOVA (Tukey's test). ns, non-significant, * $p < 0.05$, ** $p < 0.01$, *** $p < 0.001$, **** $p < 0.0001$. DT mono (control), DT monoculture; DT co, DT co-culture; DT co + Edicotinib, DT co-culture with THP-1 exposed to Edicotinib.

Table S5. Overview of the immunohistochemistry data analysis in patients' samples

Patient Number	Primary tumour		Post-treatment sample (Recurrence or curative surgery)		Response (end of follow-up)
	CD68	CD163	CD68	CD163	
P1	+ + +	+ + +	NA	NA	Responders
P2	+ + +	+ + +	NA	NA	Responders
P3	+ + +	+ + +	+ + +	+ + +	Non-responders
P4	+ + +	+ + +	+ + +	+ + +	Non-responders
P5	+ + +	+ + +	NA	NA	Non-responders
P6	NA	NA	+ + +	+ + +	Responders
P7	+ + +	+ + +	NA	NA	Non-responders
P8	NA	NA	+ + +	+ + +	Responders
P9	+ + +	+ + +	NA	NA	Responders
P10	+ + +	+ + +	NA	NA	Non-responders
P11	+ + +	+ + +	NA	NA	Non-responders

CD68 and CD163 expression are represented in symbols (+, low positive expression; ++, moderate positive expression; +++, elevated expression. All markers were analysed by the same expert pathologist. NA, not analysed

Table S6. Antibodies and conditions used in Western blot, immunohistochemistry and flow cytometry.

Technology	Antibody	Brand	Clone	Catalog number	Conditions – incubation time	Visualisation/analysis method
Western blot	SPRY4	Abcam	EPR12127	ab176337	1:5000 - Overnight	-
	ERK1/2	RayBiotech	-	144-64587-50	1:2000 - Overnight	-
	pERK1/2	BD Biosciences	-	612358	1:500 - Overnight	-
	β-actin	Sigma-Aldrich	AC-15	A5441	1:5000 - 1h	-
	α-tubulin	Sigma-Aldrich	B-5-1-2	T5168	1:4000 - 1h	-
Immunohistochemistry	CD68	VENTANA	KP1	790-2931	32`CC1 - 12`ARIO PD	OPTIVIEW
	CD163	CELL MARQUE	MRQ-26	760-4437	40`CC1 - 20`ARIO PD	OPTIVIEW
	PD-L1	DAKO	22C3	M3653	64`CC1 - 68`ARIO 1:30	OPTIVIEW
Flow cytometry	CD80	Biolegend	2D10	305220	1:100 – 15 min	BD Accuri™ C6 Plus Flow Cytometer
	CD163	Biolegend	GHI/61	333610	1:100 – 15 min	BD Accuri™ C6 Plus Flow Cytometer
